# Bile acid signaling in health and disease

**DOI:** 10.1186/s43556-026-00603-4

**Published:** 2026-10-02

**Authors:** Zhezhe Fan, Mengyi He, Taiping Liang, Bili Zhang, Na Zhao, Pan Li

**Affiliations:** 1 Department of Anesthesiology, Changzheng Hospital, Naval Medical University, Shanghai, 200003 China; 2 Department of Cardiology, Changhai Hospital, Naval Medical University, 168 Changhai Rd, Shanghai, 200433 China; 3 Department of Vascular Surgery, Changhai Hospital, Naval Medical University, 168 Changhai Rd, Shanghai, 200433 China; 4 School of Anesthesiology, Naval Medical University, 800 Xiangyin Road, Shanghai, 200433 China; 5 Department of Anesthesiology, First Medical Centre, Chinese People’s Liberation Army (PLA) General Hospital, Beijing, 100853 China

**Keywords:** Bile acids, Hydrophilicity, Bile acid pool composition, FXR, TGR5

## Abstract

Bile acids (BAs), cholesterol-derived amphipathic metabolites which are traditionally regarded as digestive surfactants, are now recognized as pleiotropic signaling molecules that act through ligand-, receptor-, and tissue-specific mechanisms to maintain systemic homeostasis. They exert biological functions via nuclear receptors (FXR, PXR, VDR, CAR) and membrane receptors (TGR5, S1PR2, MRGPRX4). These receptors activate transcriptional and membrane-initiated programs, including FXR–SHP/FGF19 feedback, TGR5–cAMP–PKA signaling, S1PR2–ERK/AKT/RhoA pathways, MRGPRX4-dependent Ca^2^⁺ signaling, PXR/CAR/VDR-driven detoxification responses, and non-receptor-mediated membrane and mitochondrial stress pathways. This review discusses how the diversity of BA structures, hepatic synthesis, conjugation, transportation, enterohepatic circulation, and microbiota-mediated transformation generates receptor- and tissue-specific signals. Furthermore, we summarize the physiological functions of BA signaling in lipid, glucose, and energy metabolism, intestinal epithelial integrity, mucosal immunity, neuroendocrine regulation, musculoskeletal adaptation, and renal water-salt balance. Additionally, we discuss the effects of disrupted BA signaling on cholestatic and metabolic liver diseases, alcohol-associated liver disease, hepatocellular carcinoma, inflammatory bowel disease, BA diarrhea, colorectal cancer, obesity, type 2 diabetes, atherosclerosis, and biliary tract diseases. Finally, the therapeutic strategies that modulate BA signaling, including the use of FXR and TGR5 agonists, FGF19 analogues, transporter inhibitors, enzyme-targeting methods, microbiota-based treatments, and hydrophilic BA therapies, are evaluated. By categorizing BA biology according to the production of ligands, receptor activation, tissue responses, and disease mechanisms, this review highlights BA signaling as an integrated regulatory network and a tractable therapeutic axis in health and disease.

## Introduction

Bile acids (BAs) are cholesterol-derived amphipathic molecules historically recognized as physiological detergents that support dietary lipid digestion, micelle formation, and fat-soluble nutrient absorption [1]. The identification of BA-sensing receptors, particularly the nuclear farnesoid X receptor (FXR) and membrane Takeda G protein-coupled receptor 5 (TGR5/GPBAR1), has redefined BAs as endocrine and paracrine signaling molecules regulating the enterohepatic circulation, hepatic metabolism, intestinal physiology, immune regulation and systemic homeostasis [2–5]. The biological effects of BA signaling are strictly controlled by ligand structure, local concentration, receptor expression patterns, and the tissue microenvironment. Changes in hydroxylation patterns, stereochemistry, side-chain modifications, conjugation states and microbial transformation affect the hydrophilic–hydrophobic balance of BAs, thus influencing receptor specificity, signal intensity, membrane interaction and tissue-specific biological effects [6, 7].

Previous reviews have comprehensively summarized key aspects of BA biology and signaling, including hepatic BA synthesis, enterohepatic circulation, FXR/TGR5-mediated signaling, gut microbiota–BA interactions, and emerging therapeutic strategies targeting BA pathways [8]. However, these important points are usually treated separately without a unified signal-centered model to integrate the whole functional process from BA production, directional transport, changes in gut microbiota, receptor recognition, downstream pathway activation and the formation of pathological changes to clinical treatment strategies [1, 9, 10].

In this review, we first define how structural features including hydroxylation, stereochemistry, conjugation, side-chain modification and microbial transformation determine ligand-specific signaling properties. Then, the main mechanisms affecting BA bioavailability, including hepatic synthesis, transporter-mediated circulation, intestinal absorption, enterohepatic feedback and microbial reconstruction, are introduced. Next, we comprehensively summarize receptor-dependent and non-receptor BA signaling pathways, with a dedicated focus on key receptors and membrane stress responses. Moreover, the physiological functions of BA signaling in metabolism, intestinal homeostasis, immunology, neuroendocrine regulation, musculoskeletal and renal systems are summarized, and finally, their pathologic abnormalities in liver and biliary diseases, gastrointestinal diseases, metabolic diseases, cardiovascular diseases and malignant tumors are discussed. Lastly, both the approved and emerging BA-targeted therapies are evaluated, indicating how this signal-centered framework can provide guidance for the precise treatment of various BA-related disorders.

## Structural determinants of ligand-specific BA signaling

### Classification and chemical diversity of BAs

**Fig. 1:**
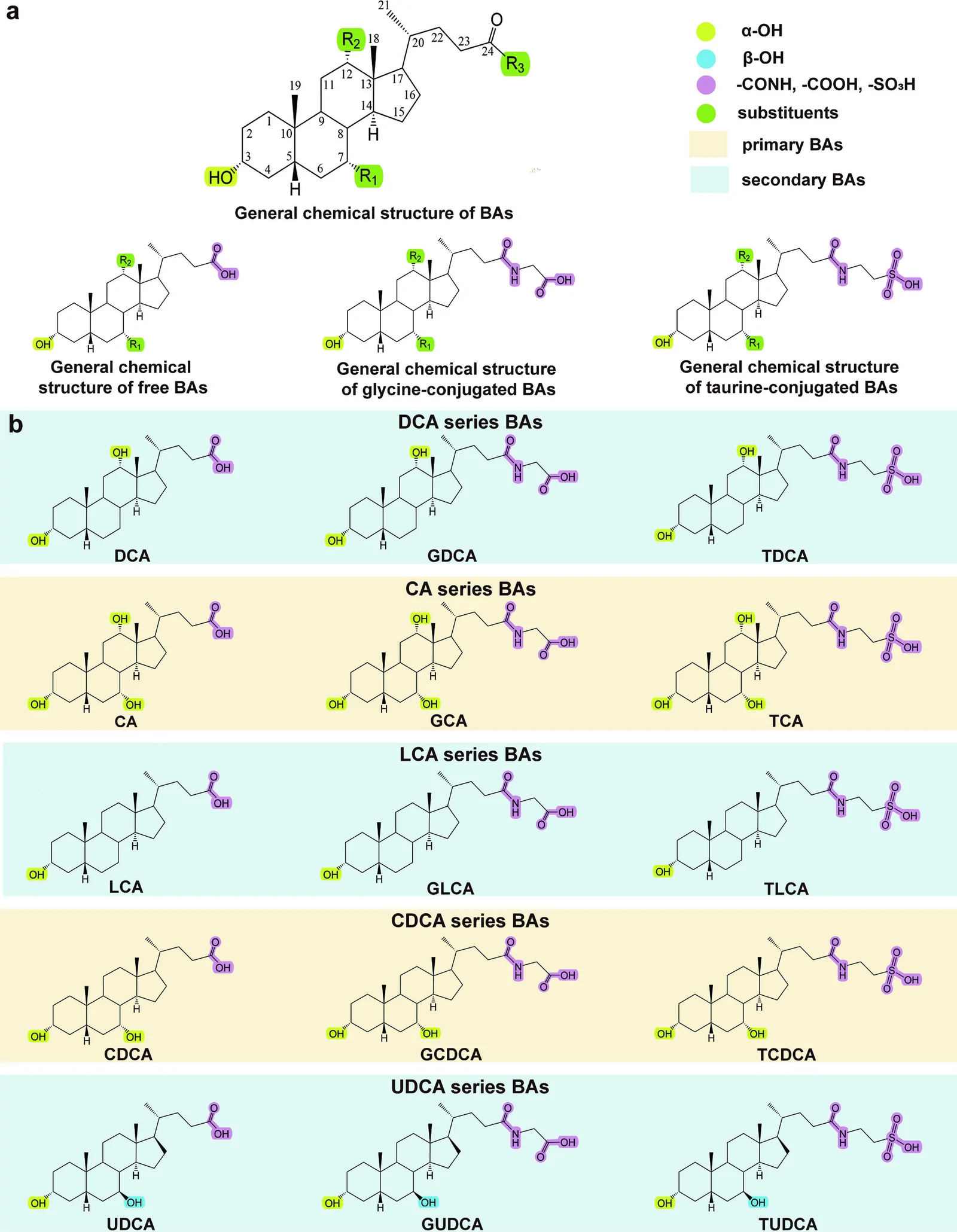
Chemical structure of BAs and the structural determinants of hydrophilicity. **a**, General chemical structure of BAs, various free and conjugated BAs. The central sterol ring with hydroxyl groups and a five-carbon side chain constitutes the general structure of BA. R₁, R₂, R₃ denote the positions of substituents. **b**, Free BAs in the human body include CA, CDCA, DCA, LCA, and UDCA. Free BAs are conjugated with glycine or taurine to form conjugated BAs. Different BAs contain corresponding hydrophilic groups: free BAs possess hydroxyl (-OH) and carboxyl (-COOH) groups, glycine-conjugated BAs contain amide bonds (-CONH), and taurine-conjugated BAs carry sulfonate groups (-SO_3_H)

BAs consist of a sterol ring and a side chain and are vital for maintaining homeostasis (Fig. 1a). As detergents for lipid absorption and signaling molecules, they integrate hepatic metabolism, intestinal physiology, and systemic homeostasis. Their structural diversity, including hydroxylation, stereochemistry, side-chain modifications, and conjugation states, significantly impacts hydrophobicity, regulating receptor binding and signal transduction. Understanding BA chemical classification is crucial for interpreting how structurally distinct BAs generate different receptor- and tissue-specific signaling outputs. BAs are classified by synthetic origin and conjugation state.

#### Synthetic origin

BAs are primarily categorized into primary and secondary BAs by synthetic origin. In humans, the liver synthesizes two primary BAs from cholesterol: cholic acid (CA) and chenodeoxycholic acid (CDCA), with a ratio of approximately 1:1. Rodents also produce muricholic acids (MCAs), which reflects species-specific diversity [11, 12]. Primary BAs are converted to secondary BAs by gut microbiota, increasing hydrophobicity and expanding signaling repertoires [13]. The microbiota can further modify BAs to form microbially conjugated BAs, enriching secondary BA diversity and potentially modulating host immunity [14–16] (Fig. 1b). Notably, the microbiome generates amino acid–conjugated BAs, including phenylalanine- and tyrosine-conjugated species (Phe-BAs, Tyr-BAs) [14].

#### Conjugation state

BAs are further divided into free and conjugated forms. Free BAs (e.g., CA, CDCA, deoxycholic acid [DCA], and lithocholic acid [LCA]) lack amino acid conjugation, are highly cytotoxic, and are passively absorbed in the intestine [17] (Fig. 1b). Conjugated BAs, formed by linking glycine or taurine in the liver, have reduced pKa values, enhancing water solubility, secretion efficiency, and membrane protection. Conjugation patterns vary among species, reflecting dietary and evolutionary adaptations [12].

Subtle variations in BA stereochemistry, hydroxylation patterns, conjugation status, and side-chain modifications shape their hydrophilic–hydrophobic gradient, thereby influencing cytotoxicity, membrane interactions, and receptor activation. For example, ursodeoxycholic acid (UDCA) is relatively hydrophilic, while LCA, DCA, and CDCA are highly hydrophobic. These differences profoundly affect BA binding to receptors like FXR and TGR5, modulating downstream signaling. Overall, the order of hydrophobicity is LCA > DCA > CDCA > CA > UDCA, with conjugated BAs being more hydrophilic than free forms. Thus, the structural features of BAs contribute to ligand-specific receptor activation, non-receptor membrane effects, and physiological outcomes.

### Structural determinants of BA hydrophobicity

The structure of BAs affects not just classification but also hydrophobicity. The hydrophilicity of BAs, a critical determinant of their biological activity and signaling behavior, is mainly governed by the number and configuration of hydroxyl groups and the conjugation state of the side-chain carboxyl group [18–20].

#### The number and configuration of hydroxyl groups

The number and spatial arrangement of hydroxyl (-OH) groups on the sterol ring system determine BA hydrophilicity by enabling hydrogen bonding. Generally, more -OH groups increase hydrophilicity, while fewer increase hydrophobicity. LCA is described as having only one hydroxyl group at C3, which contributes minimally to hydrophilicity, making LCA the most hydrophobic among major human BAs. The configuration of these hydroxyl groups (α or β orientation) further modulates hydrophobicity. Most natural BAs have α-oriented hydroxyl groups, forming a polar face and a hydrophobic side, crucial for amphiphilicity. Notably, UDCA has a β-oriented C7-OH group, enhancing solubility despite sharing the same -OH count as CDCA [19, 21].

#### The conjugated state of the carboxyl group

The side-chain structure of BAs also affects hydrophilicity. Natural BAs have a C24 carboxylic acid group that conjugates with glycine or taurine, forming conjugated BAs. This process lowers pKa, enhances water solubility, and reduces cytotoxicity [1]. Glycine conjugation adds one amide bond, increasing hydrophilicity; taurine conjugation adds both an amide bond and a sulfonate group, which is highly polar and negatively charged at physiological pH, making taurine-conjugated BAs the most hydrophilic. The functional groups attached to the five-carbon side chain during conjugation determine hydrophilicity [22]. By altering solubility, transport behavior, and receptor accessibility, conjugation also changes the location and intensity of BA signaling.

### Structure–activity relationships in BA signaling

BAs are cholesterol-derived metabolites that act as critical signaling molecules, regulating lipid metabolism, glucose homeostasis, and immune responses [1]. Their biological activity depends on structural features, including the number and position of hydroxyl groups, stereochemistry, side-chain modifications, conjugation state, and hydrophobicity [23, 24]. These factors dictate interactions with receptors and cellular targets, influencing signal intensity and outcomes. Therefore, BA structure provides an upstream explanation for why different BA ligands produce distinct signaling outputs even within the same receptor family.

#### Impact of structural factors on receptor binding affinity

BA receptors, such as FXR and TGR5, exhibit high ligand-structure selectivity. Their hydrophobic pockets recognize hydroxyl groups via hydrogen bonds, making hydroxyl number, position, and stereochemistry crucial for binding. For example, CDCA activates FXR more effectively than CA or DCA due to stable interactions in the FXR pocket [25, 26], while CA's extra C12 hydroxyl causes steric clashes, reducing binding. Hydrophobicity also matters: hydrophobic BAs such as LCA and DCA strongly activate TGR5, likely through interactions with its transmembrane domain [27].

#### BAs as full agonists, partial agonists, and antagonists

BAs exhibit distinct functional effects on the same receptor, depending on their ability to stabilize receptor conformations. Full agonists (e.g., CDCA) fully activate receptors, promoting co-activator binding and downstream gene expression [28, 29]. Partial agonists, like CA, partially stabilize the activated conformation, resulting in weaker signaling. Additionally, gut microbiota-derived modifications alter BA composition, influencing receptor activity. For instance, T-βMCA, a microbiota-modified BA, acts as a natural FXR antagonist, inhibiting FXR-dependent gene expression [30, 31]. The current BA classification framework (full/partial agonists, antagonists), mostly based on in vitro single-pathway data, lacks unified potency criteria, overlooks multi-receptor cross-activation, in vivo metabolic dynamics, and microbiota modification effects; and cannot fully explain the efficacy contradictions in clinical translation.

## Regulation of BA signaling exposure by synthesis, transport, and microbial remodeling

### BA synthesis, transport and enterohepatic circulation

**Fig. 2:**
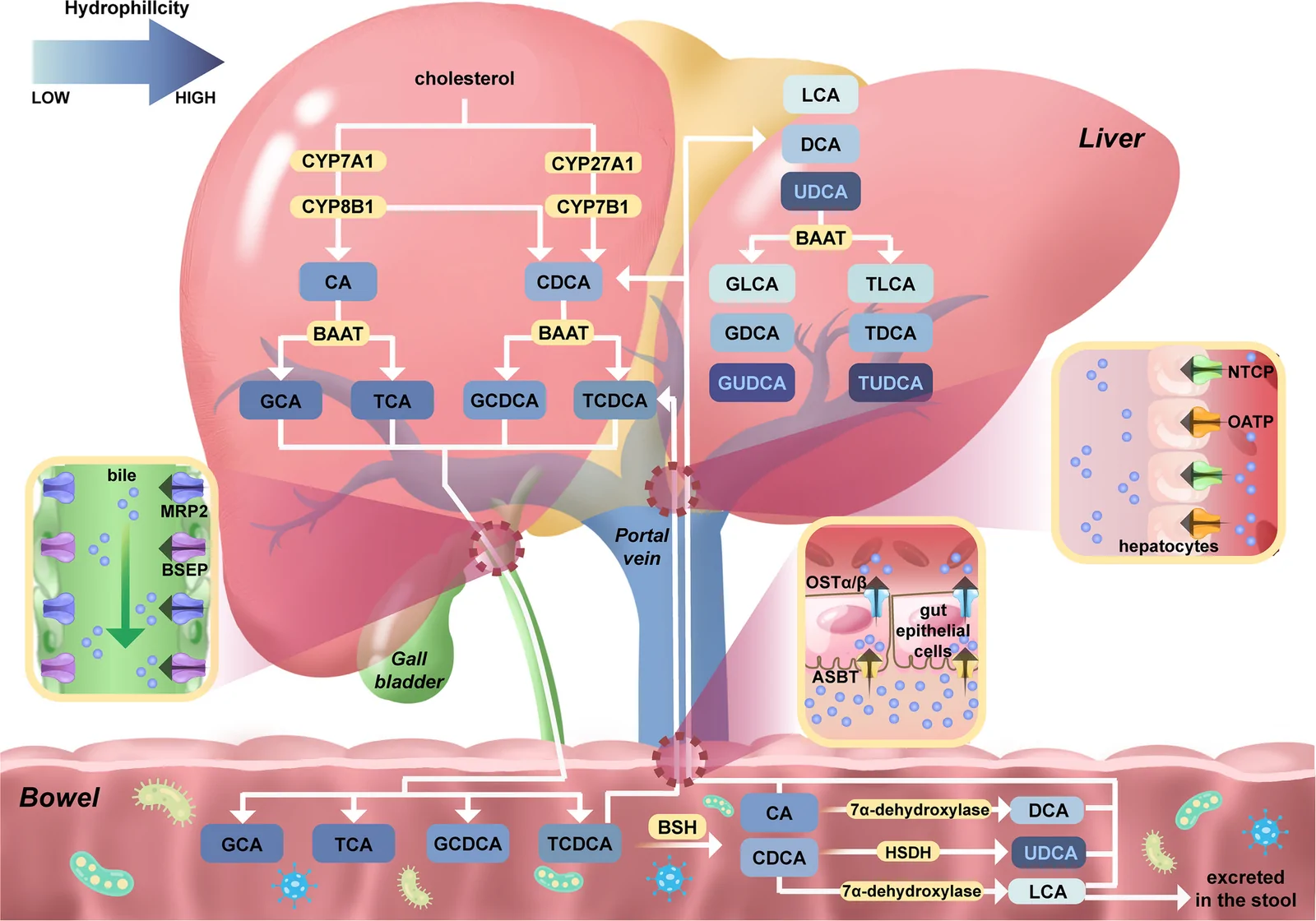
In vivo hydrophilicity regulation of BAs. The figure illustrates the enterohepatic circulation process of BAs, including their synthesis and transport. These two processes are closely related to the hydrophilicity regulation of BAs, primarily corresponding to enzyme-mediated metabolic regulation and transporter-mediated transport regulation. The changes in hydrophilicity of BAs are represented by gradient colors, with darker shades indicating stronger hydrophilicity

In hepatocytes, cholesterol is converted to primary BAs via the classical (using [cholesterol 7α-hydroxylase] CYP7A1 and [sterol 12α-hydroxylase] CYP8B1) and alternative (using [sterol 27-hydroxylase] CYP27A1) pathways [13, 32]. Newly synthesized primary BAs are activated by bile acyl-CoA synthetase (BACS) and conjugated with glycine or taurine by BA-CoA: amino acid N-acyltransferase (BAAT), generating conjugated BAs. These conjugates are mainly secreted into bile via the bile salt export pump (BSEP), with multidrug resistance-associated protein 2 (MRP2) also aiding in biliary transport, and stored in the gallbladder before being released into the intestine after meals. In the intestine, bacterial bile salt hydrolases (BSH) deconjugate BAs. Then, 7α-dehydroxylation turns primary BAs into secondary BAs, and hydroxysteroid dehydrogenases (HSDHs) can convert CDCA into UDCA [33, 34]. About 95% of BAs are reabsorbed, mainly in the terminal ileum via apical sodium-dependent BA transporter (ASBT), exported into portal blood via organic solute transporter complex (OSTα/β), and returned to hepatocytes via sodium-dependent taurocholate cotransporting polypeptide (NTCP) and organic anion transporting polypeptides (OATPs), maintaining enterohepatic circulation [21, 35] (Fig. 2). Through this cycle, BA ligands repeatedly pass through liver and intestinal compartments, creating receptor exposure patterns that are essential for FXR, TGR5, S1PR2, and other BA-responsive pathways.

### Enzyme-mediated regulation of BA synthesis

BA synthesis critically determines BA signal availability and ligand composition. While BAs perform essential physiological roles, their potential cytotoxicity necessitates tight regulation of synthesis rates and proportions through multi-level control of key enzyme activities. This ensures that receptor activation remains within a physiological range. In the classical synthesis pathway, CYP7A1 serves as the main rate-limiting enzyme, initiating synthesis by converting cholesterol to 7α-hydroxycholesterol. Elevated BA levels activate FXR, which suppresses CYP7A1 transcription via a negative feedback loop [36]. Additionally, intestinal fibroblast growth factor 19 (FGF19; FGF15 in mice) inhibits CYP7A1 expression through hepatic FGF receptor 4 (FGFR4)–β-Klotho signaling [37, 38].

CYP8B1 regulates the proportion of CA produced, affecting total BA synthesis, the CA/CDCA ratio, the BA pool's structure and the hydrophilic-hydrophobic balance [39]. In the alternative pathway, CYP27A1 initiates CDCA synthesis, normally contributing only minimally but compensating when the classical pathway is impaired in some disease states [40, 41]. Enzymes like CYP7B1, CYP3A4, and certain HSDHs or reductases are also involved in BA metabolism [42–45]. Recent studies show BA synthesis enzymes are regulated not only by BA feedback but also by metabolic signals (e.g., insulin, inflammatory cytokines, and nutritional status) [46–49].

### Transporter-mediated regulation of BA distribution

The tissue exposure of BAs is regulated by their synthesis rate and transmembrane transporters in the liver, intestine, and kidney, as BAs require specific transport systems to cross cell membranes. These transporters are crucial for enterohepatic circulation, controlling BA redistribution, pool size, composition, concentration gradients, and BA signaling. In the liver, BAs enter hepatocytes mainly via basolateral NTCP and OATPs [50–52]. When BA levels rise, they activate FXR signaling, which then inhibits uptake pathways such as NTCP to prevent excessive BA accumulation in hepatocytes. On the canalicular membrane, BSEP transports BAs into bile, and its dysfunction can lead to cholestatic liver diseases [53–55]. Transporters such as MRP2 also aid bile secretion, influencing BA excretion and composition [56–58].

Intestinal BA reabsorption is critical for maintaining the BA pool. The ileal ASBT on epithelial cells actively moves conjugated BAs from the intestinal lumen into cells, starting enterohepatic circulation. Impaired ASBT function reduces BA reabsorption, increases fecal excretion, and reduces the BA pool size [59, 60]. Once inside intestinal cells, BAs enter the portal vein via the basolateral OSTα/β, regulated by the BA-sensing nuclear receptor FXR and its downstream FGF19 signaling [61]. In cholestasis, alternative transporters help remove BAs from hepatocytes to reduce toxicity. Previous findings show that preclinically potent ASBT/OSTα/β inhibitors often lose efficacy in human trials. This systemic clinical translation failure may arise from long-term oversimplification of BA transport regulatory networks and insufficient recognition of the modulatory roles of the gut microbiota microenvironment.

### Microbiota-driven remodeling of the BA pool

The gut microbiota plays a key role in remodeling BA ligands and shaping BA signaling exposure. It converts primary BAs into secondary BAs, changing receptor agonist or antagonist profiles and reshaping pool abundance and composition. This is achieved by influencing recovery efficiency, fecal excretion, intestinal feedback intensity, and local BA burden. An important initial step in this process is deconjugation. Bacteroides, Clostridium, and Lactobacillus are among the gut bacteria capable of performing deconjugation. Deconjugation alters the solubility and ionization of BAs, changing their chemical form and intestinal recovery kinetics [62]. This activity makes some BAs more likely to undergo further transformation or be excreted distally, thereby altering BA ligand composition and downstream signaling [63].

After deconjugation, a subset of strictly anaerobic bacteria, such as Clostridium scindens and Clostridium hylemonae, can mediate the 7α-dehydroxylation reaction, further converting primary BAs into secondary BAs [64, 65]. Secondary BAs have strong receptor activity and can alter the hydrophobicity, intestinal distribution, and enterohepatic circulation efficiency of BA pool molecules. Some secondary BAs are reabsorbed and recycled, whereas those that are highly hydrophobic and toxic are excreted after liver detoxification [66]. The microbiota reshapes BA signaling mainly by changing the ligand composition sensed by ileal FXR, affecting FGF15/FGF19-mediated hepatic negative feedback and regulating host BA synthesis. Different BAs have varying effects on FXR activation or inhibition. For example, in germ-free mice, certain FXR-antagonistic BAs increase; colonization with normal microbiota reduces their abundance, relieving ileal FXR inhibition, enhancing FGF15 expression, and reducing BA synthesis [30]. Current microbiota-driven BA remodeling studies rely heavily on simplified germ-free or single-strain systems and largely overlook the redundancy and dynamic regulation of the human gut microbiota; consequently, microbiota-targeted interventions cannot achieve precise steady-state BA ligand regulation in heterogeneous populations.

## BA-activated signaling pathways

Upstream regulation of BA ligand production, transport, and microbial remodeling determines the activation mode and biological output of downstream signaling pathways. For example, FXR–SHP–CYP7A1 negative feedback and transporter-mediated enterohepatic circulation regulate the magnitude and location of BA exposure, whereas microbial deconjugation and 7α-dehydroxylation alter ligand structure and receptor preference. Together, these processes connect BA chemistry and tissue distribution with receptor activation, cellular stress responses, and disease phenotypes [7, 67, 68]. Among the major BA-responsive pathways, FXR, TGR5, and VDR have been extensively studied [35, 69].

### Nuclear receptor–mediated signaling

BAs can bind to nuclear receptors, which is the core mechanism of BA signaling regulation. Nuclear receptors are widely expressed and regulate target gene transcription to participate in BA metabolism.

#### FXR signaling

**Fig. 3:**
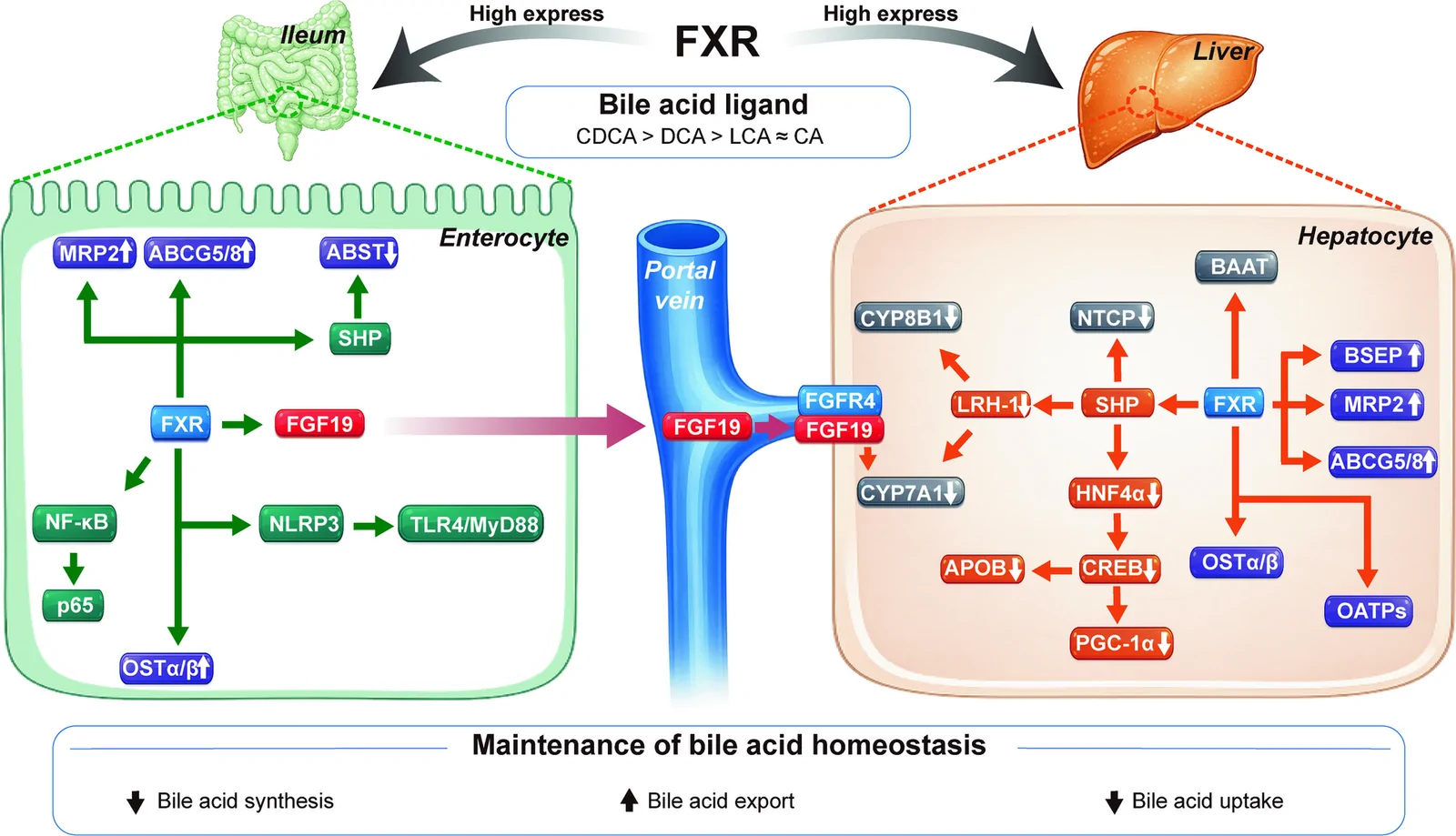
Enterohepatic FXR signaling coordinates BA synthesis, transport, and homeostatic adaptation. BAs act as endogenous ligands for FXR, with relative agonistic potency generally following the order CDCA > DCA > LCA ≈ CA. In the ileum, FXR activation in enterocytes induces FGF19 secretion and regulates BA transporters, including ASBT, organic solute transporter α/β (OSTα/β), MRP2, and ABCG5/8, while also modulating inflammatory pathways such as NF-κB and NLRP3 inflammasome signaling. Intestinally derived FGF19 reaches the liver through the portal circulation and activates the hepatic FGFR4 pathway, thereby reinforcing feedback inhibition of BA synthesis. In hepatocytes, FXR induces SHP, which suppresses LRH-1- and HNF4α-dependent transcriptional programs to downregulate CYP7A1 and CYP8B1, reducing de novo BA synthesis. FXR also promotes canalicular and basolateral BA efflux through BSEP, MRP2, ABCG5/8, and OSTα/β, while limiting hepatic BA uptake through NTCP repression. Together, intestinal and hepatic FXR signaling forms an integrated enterohepatic feedback network that decreases BA synthesis, enhances BA export, restricts BA uptake, and preserves BA pool homeostasis

FXR is a pivotal BA-sensing nuclear receptor for maintaining BA homeostasis [49]. When BA levels increase, FXR initiates a hepatobiliary negative feedback network that inhibits de novo BA synthesis [1]. Among the BAs in the BA pool, potent agonists can activate FXR and thereby translate ligand exposure into transcriptional responses. Once activated, FXR targets specific genes to suppress BA synthesis, promote BA efflux and transport, and limit hepatocyte damage [70, 71]. Given its central position at the interface of BA sensing, enterohepatic communication, and adaptive transporter regulation, FXR represents the prototypical nuclear receptor pathway through which BA signals are actively maintained (Fig. 3).

The classic mechanism of FXR signaling operates as follows: FXR activates the small heterodimer partner (SHP). Subsequently, SHP suppresses the transcriptional activity of liver receptor homolog-1 (LRH-1) and hepatocyte nuclear factor 4α (HNF4α) on CYP7A1 [36]. Through this process, CYP7A1, the key enzyme for total BA synthesis, is indirectly inhibited via SHP [72]. Beyond the FXR–SHP–CYP7A1 axis, there are both SHP-dependent and independent pathways. The intestinal FGF15/19–FGFR4 pathway and intrahepatic SHP jointly form a multi-layered negative feedback loop for BA synthesis [73–75]. Specifically, intestinal FXR induces the production of FGF15/19, which then inhibits CYP7A1 and regulates CYP8B1 in the liver, creating a cross-organ system [37]. Recent studies have indicated that FXR may utilize additional FGF-like pathways for BA negative feedback. Among them, the FGF19 pathway is of great significance for BA regulation and holds therapeutic potential for metabolic disorders and cholestasis [49, 76, 77].

The induction of BSEP expression by FXR is vital for reducing the hepatic BA load. When activated, FXR forms a heterodimer with the retinoid X receptor (RXR). This heterodimer then binds to the FXR response elements in the BSEP promoter, directly promoting BSEP transcription [78]. When the intracellular BA levels rise, FXR inhibits BA synthesis and promotes its efflux via BSEP. In the ileum, FXR induces the expression of ileal BA-binding protein (IBABP), OSTα, and OSTβ. These proteins are responsible for intracellularly transporting BAs and basolaterally effluxing them into the portal circulation, thereby reducing BA retention and enabling efficient recycling [79, 80]. Moreover, FXR indirectly inhibits BA uptake genes (e.g., NTCP) at hepatocyte membranes via intermediaries like SHP [81], mainly through indirect negative feedback. In summary, when BAs are in excess, FXR reduces the BA load by inhibiting synthesis, enhancing efflux, and limiting reuptake.

#### PXR signaling

Unlike FXR, pregnane X receptor (PXR) acts as a defense receptor against high-risk, hydrophobic, and cytotoxic BAs like LCA. Activated PXR initiates a "Phase I Oxidation-Phase II Conjugation-Phase III Efflux" detoxification process. In contrast to FXR's role in optimizing BA circulation, PXR mainly focuses on reducing BA toxicity. CYP3A4, a key PXR target gene, is induced by LCA to alleviate BA toxicity. Mechanistically, PXR forms a heterodimer with the RXR. This heterodimer then binds to PXR response elements in the regulatory region of the CYP3A gene, promoting its transcription [82]. CYP3A4 metabolizes hydrophobic BAs via hydroxylation, reducing their toxicity.

During Phase II metabolism, several enzymes play crucial roles in BA detoxification. Sulfotransferase family 2 A member 1 (SULT2A1), a major BA-sulfating enzyme, sulfates BAs to increase their hydrophilicity and reduce toxicity. Meanwhile, the UDP-glucuronosyltransferase (UGT) family, including UGT1A3 and UGT2B7, aids in BA clearance via glucuronidation. These enzymes, sharing LCA as a substrate, are regulated by PXR [66]. In terms of Phase III efflux, PXR also enhances BA efflux via transporters like MDR1/ABCB1 and MRP3/MRP4. The upregulation of MRP3/MRP4 serves as a protective response in cholestasis [83, 84]. MRP4 can transport sulfated BAs, but its regulation also involves the CAR, nuclear factor erythroid 2-related factor 2 (Nrf2), and BA self-induction [85, 86], indicating PXR is not the only factor involved in efflux adaptation. Overall, PXR drives a detoxification program focused on clearance, acting as a crucial second-line defense against hydrophobic and toxic BA imbalances.

#### VDR signaling

The VDR, the vitamin D receptor, can be activated by LCA and its metabolites, linking BA-derived ligands to transcriptional programs for detoxification, intestinal defense, and BA homeostasis. Once activated, VDR regulates genes involved in BA synthesis and excretion. Activated VDR reduces BA synthesis by inhibiting CYP7A1 transcription [87, 88]. It also cross-regulates with FXR and liver X receptor α (LXRα) to modulate BA synthesis, distribution, and hydrophobicity, notably by inhibiting LXRα signaling [87]. In the intestine and liver, VDR modifies BA distribution by upregulating ASBT and OSTα/β [89]. Moreover, VDR deficiency is linked to lower total BA levels, especially CDCA and its metabolites [90].

When activated by LCA, VDR induces the expression of CYP3A4/CYP3A, crucial for local LCA detoxification in the intestinal epithelium [91]. In intestinal models, LCA-induced CYP3A4 induction heavily relies on VDR [92]. Loss of VDR in mice worsens liver injury, and this can be partially reversed by restoring intestinal CYP3A4 [93]. VDR also transcriptionally induces SULT2A1, promoting BA sulfation, especially that of LCA [94]. Although there is an overlap with PXR-mediated detoxification, VDR places more emphasis on intestinal barrier defense. The LCA–VDR–SULT2A1 pathway acts as a localized innate response against toxic BAs in the gut. Activated VDR induces genes involved in BA detoxification and transcriptional programs for BA homeostasis [95]. Additionally, VDR regulates antimicrobial peptides and immune molecules, linking the intestinal microbiota, BAs, and mucosal immunity [10]. However, current VDR–BA homeostasis research has not fully clarified how VDR's response to BA signals differs across tissues (e.g., intestine vs. liver) and how it changes over time.

#### CAR signaling

CAR signaling, similar to PXR, induces drug-metabolizing enzymes and transporters and plays a more critical role under stress conditions, such as cholestasis. When FXR/SHP is impaired, CAR/PXR is activated to enhance the metabolism and efflux of BAs and bilirubin, thereby reducing liver injury. Studies on progressive familial intrahepatic cholestasis type 5 (PFIC5) show that FXR/SHP deficiency triggers the activation of CAR/PXR–CYP2B/CYP2C, which aids in bilirubin clearance and liver protection [96]. CAR targets CYP2B, CYP2C, parts of CYP3A, and phase II enzymes like UGT1A1 and SULT2A1, facilitating the conjugation of bilirubin and BAs. CAR activation boosts bilirubin clearance, reducing serum bilirubin levels [97]. In BA toxicity models, the protective effect of CAR is linked to enhanced SULT activity and BA sulfation [98]. CAR also regulates efflux transporters like MRP2, MRP3, and MRP4. In cholestasis, MRP4 is upregulated to excrete sulfated BAs and reduce hepatotoxicity [99, 100]. Although CAR and PXR collaborate, their roles vary. In summary, nuclear receptor-mediated BA signaling forms a complex network. FXR regulates BA pool size and composition, while PXR and CAR detoxify hydrophobic BAs, and VDR protects the intestine from LCA. Initially, elevated BAs activate FXR. As toxicity increases, the defense mechanisms of PXR, CAR, and VDR are activated to prevent BA-induced injury.

### G protein-coupled receptor signaling

BA signaling via G protein-coupled receptors (GPCRs) has two main features. Firstly, it enables rapid, context-specific membrane transduction, which regulates second messengers and ion channels within minutes to hours. Secondly, it shows high sensitivity to the physicochemical properties of BAs, such as hydrophobicity, which influence receptor activation and downstream signals. Key GPCRs like TGR5 (involved in metabolic regulation), S1PR2 (in hepatobiliary signal changes), and MRGPRX4 (in sensory neurons) indirectly affect transcription factors and chromatin via specific pathways, causing diverse effects.

#### TGR5 signaling

**Fig. 4:**
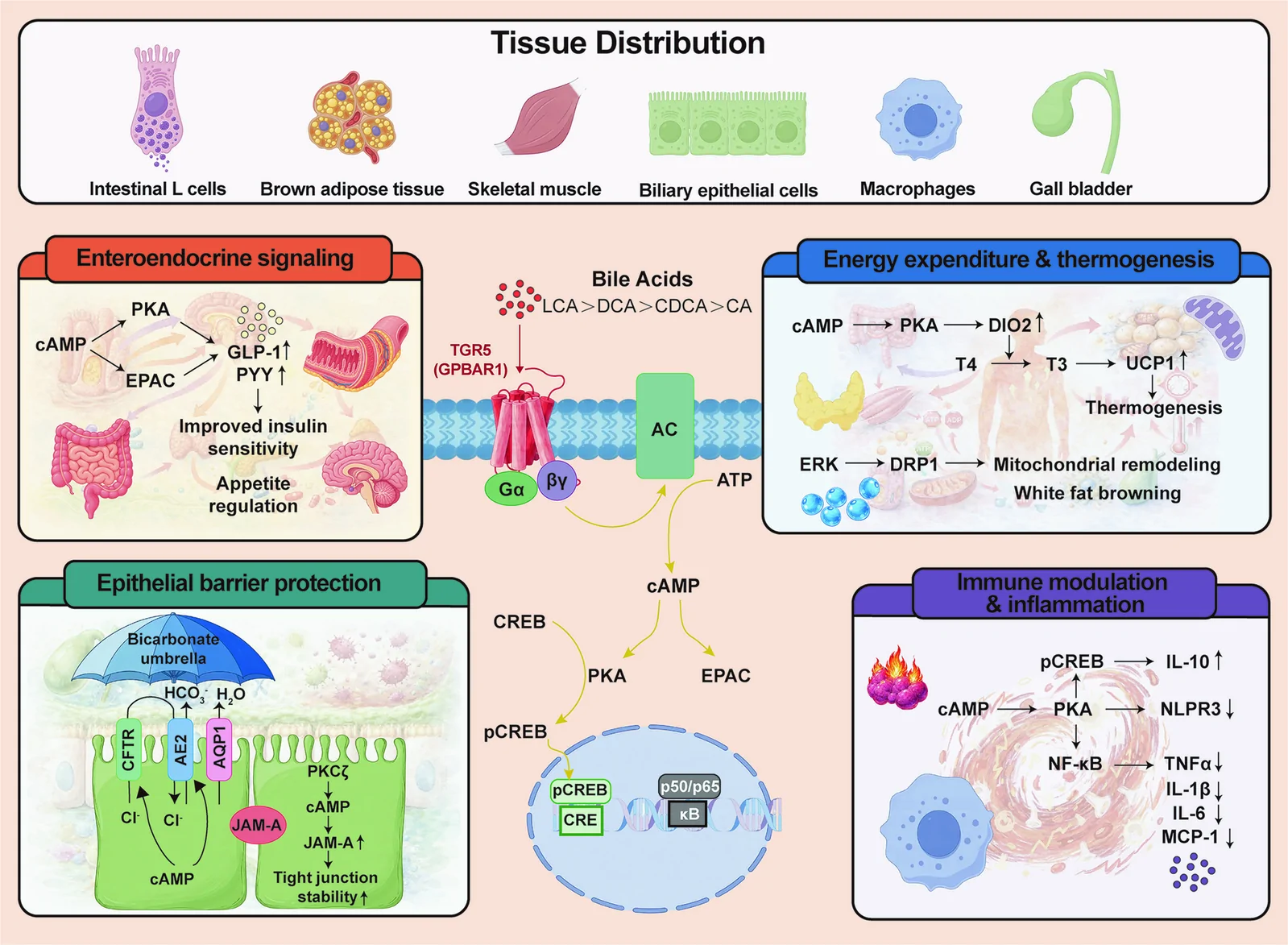
TGR5 signaling mediates BA–dependent metabolic, epithelial, and immune regulation across tissues. TGR5 is broadly expressed in intestinal L cells, brown adipose tissue, skeletal muscle, epithelial cells, macrophages, and the gallbladder. BAs activate TGR5 with potency LCA > DCA > CDCA > CA, triggering Gαs–adenylyl cyclase signaling and increasing intracellular cAMP. Downstream activation of PKA, EPAC, and CREB drives tissue-specific responses. In enteroendocrine cells, TGR5 promotes GLP-1 and PYY secretion, improving insulin sensitivity and appetite control. In metabolic tissues, it enhances thermogenesis and energy expenditure via the cAMP–PKA–DIO2–T3–UCP1 axis and promotes mitochondrial remodeling. In epithelial cells, TGR5 strengthens barrier integrity by inducing bicarbonate secretion and stabilizing tight junctions. In macrophages, TGR5 exerts anti-inflammatory effects by activating CREB-dependent IL-10 expression and suppressing NF-κB and NLRP3 signaling. Collectively, TGR5 links BA composition to systemic metabolic and immune homeostasis

TGR5 can be activated by both free and conjugated BAs and acts as a membrane sensor that translates BA ligand exposure into metabolic and inflammatory responses [67]. Among unconjugated BAs, the activation potency follows the order of LCA > DCA > CDCA > CA > UDCA. Secondary BAs and their conjugates are potent agonists, suggesting that TGR5 is especially sensitive to ligand hydrophobicity and conformation rather than concentration alone. Its main signaling pathway is the Gs–adenylyl cyclase–cAMP–PKA pathway, which activates downstream effectors like exchange protein directly activated by cAMP (EPAC), ERK1/2, and AKT, with cell type-specific functions. However, TGR5 exhibits a dual role. Systemic activation of TGR5 improves glucose and energy metabolism but may cause adverse effects such as gallbladder filling, raising tissue-specific safety concerns [101, 102]. These issues limit the clinical use of TGR5 agonists. Although selective agonists like SB-756050 have entered early-phase trials for type 2 diabetes, long-term safety and tissue targeting remain key challenges for therapy development [103] (Fig. 4).

#### S1PR2 signaling

S1PR2, a GPCR for sphingosine-1-phosphate (S1P), also responds to conjugated BAs like TCA in the hepatobiliary system. It is expressed in hepatocytes, cholangiocytes, and macrophages and couples with Gi/o, Gq/11, and G12/13 to activate the ERK1/2, AKT, and RhoA/ROCK pathways [104–106]. A key mechanism is S1PR2-mediated upregulation of sphingosine kinase 2 (SphK2), which increases nuclear S1P levels, inhibits histone deacetylases 1/2 (HDAC1/2), and enhances histone acetylation to remodel the expression of metabolism-related genes. Conjugated BAs specifically activate this axis, as shown in S1PR2 or SphK2 knockout models [107]. Beyond metabolic regulation, S1PR2 contributes to disease pathogenesis. In cholangiocarcinoma (CCA), TCA/S1PR2 signaling promotes invasive growth by upregulating cyclooxygenase-2 (COX-2) and prostaglandin E2 [108]. In cholestasis and macrophage models, S1PR2 drives the activation of the NLRP3 inflammasome, leading to inflammation and fibrosis [105]. However, how this ligand-specific signaling dynamically interacts with other BA-sensing receptors to shape disease outcomes remains poorly characterized.

#### MRGPRX4 signaling

MAS-related G protein-coupled receptor X4 (MRGPRX4), a human Mas-related GPCR, is a key sensory receptor for cholestatic pruritus and is selectively expressed in dorsal root ganglion neurons. It can be directly activated by BAs, triggering Ca^2^⁺ influx and itch via the Gq/11–PLCβ–IP3/DAG–Ca^2^⁺ pathway [109, 110]. Unlike in mice, where TGR5 drives BA-induced itch, human TGR5 is located in satellite glial cells, which limits the relevance of rodent models. Structural studies have highlighted 3-sulfated BAs as critical endogenous agonists, with modifications at the 3-position/3-OH being essential for MRGPRX4 recognition. This reveals species-specific BA signaling: MRGPRX4 causes human pruritus through unique mechanisms, and the chemical properties of BAs (e.g., sulfation) determine receptor specificity [7], underscoring the need for human-targeted therapies for cholestatic itch.

In general, TGR5, S1PR2, and MRGPRX4 mediate different BA GPCR signaling pathways. TGR5 binds hydrophobic secondary BAs to regulate metabolism and bile secretion; S1PR2 activates ERK/AKT pathways via conjugated BAs, driving hepatobiliary inflammation; MRGPRX4 converts abnormal BA signals into human-specific itch through sensory neurons. The structural features of BAs affect receptor selectivity, while local BA exposure determines pathway activation in specific tissues, linking BA dynamics to the progression of liver diseases.

### Non-receptor–mediated BA signaling

BAs exert non-receptor effects due to their amphipathic/hydrophobic nature. Highly hydrophobic BAs (e.g., DCA, CDCA, and LCA) disrupt plasma and intracellular membranes, altering cholesterol distribution and displacing membrane proteins. This triggers Ca^2^⁺ overload, membrane depolarization, ROS-driven NF-κB/p38 MAPK activation, mitochondrial membrane potential collapse, and metabolic dysfunction, collectively promoting cellular stress, inflammation, and apoptosis [111–114]. Conversely, hydrophilic BAs such as UDCA and TUDCA protect cells by activating the PI3K–Akt pathway to inhibit mitochondrial apoptosis and by suppressing endoplasmic reticulum stress (ERS) sensors (e.g., PERK, ATF6, IRE1, and GRP78) and downstream apoptotic signaling, thereby reducing inflammation and cell death [115, 116]. Thus, BA hydrophobicity dictates membrane-perturbing capacity and functional outcomes: hydrophobic BAs induce stress, whereas hydrophilic BAs act as protectors (Table 1). In essence, hydrophobic BAs exhibit a concentration-dependent dual nature: at physiological levels, they facilitate membrane remodeling, receptor crosstalk, motility, and wound repair; beyond a threshold set by local BA exposure, the same species induce genotoxicity, apoptosis, ROS, and inflammation. Conjugation and stereochemistry modulate this threshold, determining whether a given BA signaling environment supports homeostasis or pathology.

**Table 1:** Non-receptor–mediated BA signaling crosstalk

Signaling mechanism	Representative BAs	Molecular targets	Mechanism	Biological effects	Representative references
Membrane perturbation and lipid raft remodeling	DCA, CDCA	membrane cholesterol, lipid rafts	BAs insert into lipid bilayers and alter membrane microdomain organization, affecting membrane protein clustering and signaling platforms	Modulation of receptor signaling, cell stress responses	[117, 118]
Ca^2^⁺ signaling and ion channel modulation	CA, DCA, CDCA	Ca^2^⁺ channels, Na⁺/Ca^2^⁺ exchanger (NCX)	BAs alter intracellular Ca^2^⁺ handling and membrane depolarization, affecting ion currents and electrophysiology	Changes in contractility, arrhythmia, altered cell signaling	[111, 112, 119, 120]
Membrane perturbation–mediated epidermal growth factor receptor (EGFR) transactivation (Ca^2^⁺-dependent)	DCA	EGFR, CaMKII, c-Src	Induces intracellular Ca^2^⁺ elevation and activates CaMKII and c-Src, leading to ligand-independent EGFR transactivation and sustained ERK1/2 signaling distinct from canonical EGF-induced receptor trafficking	Promotes proliferation and tumor-associated signaling in colon epithelial/cancer cells	[121]
Reactive oxygen species (ROS) signaling	DCA, CDCA	ROS, NF-κB, p38 MAPK	BAs stimulate ROS production, which activates inflammatory signaling pathways	Endothelial inflammation, immune activation, tissue injury	[122]
Mitochondrial dysfunction	LCA, DCA, CDCA	mPTP, mitochondrial membrane potential	Hydrophobic BAs increase mitochondrial permeability transition and disrupt oxidative metabolism	Mitochondrial damage, apoptosis, metabolic dysfunction	[123]
PI3K–Akt survival signaling	UDCA, TUDCA	Akt, Bad, Bcl-2	Activation of Akt phosphorylation suppresses mitochondrial apoptosis pathways	Cytoprotection, improved mitochondrial function	[115, 116]
ERS signaling	TUDCA	PERK, ATF6, IRE1, GRP78	TUDCA suppresses ER stress sensor activation and downstream apoptotic pathways	Reduced inflammation and apoptosis	[124, 125]
Apoptosis signaling	LCA, hydrophobic BAs	caspases, EphA2	Regulation of apoptosis-related receptors and mitochondrial pathways	Cell survival or programmed cell death depending on BA type	[126]
Inflammatory signaling	DCA	NF-κB, cytokines	BAs stimulate inflammatory cascades through oxidative stress and transcription factor activation	Chronic inflammation and tissue injury	[127]

## Physiological roles of BA signaling in systemic homeostasis

**Fig. 5:**
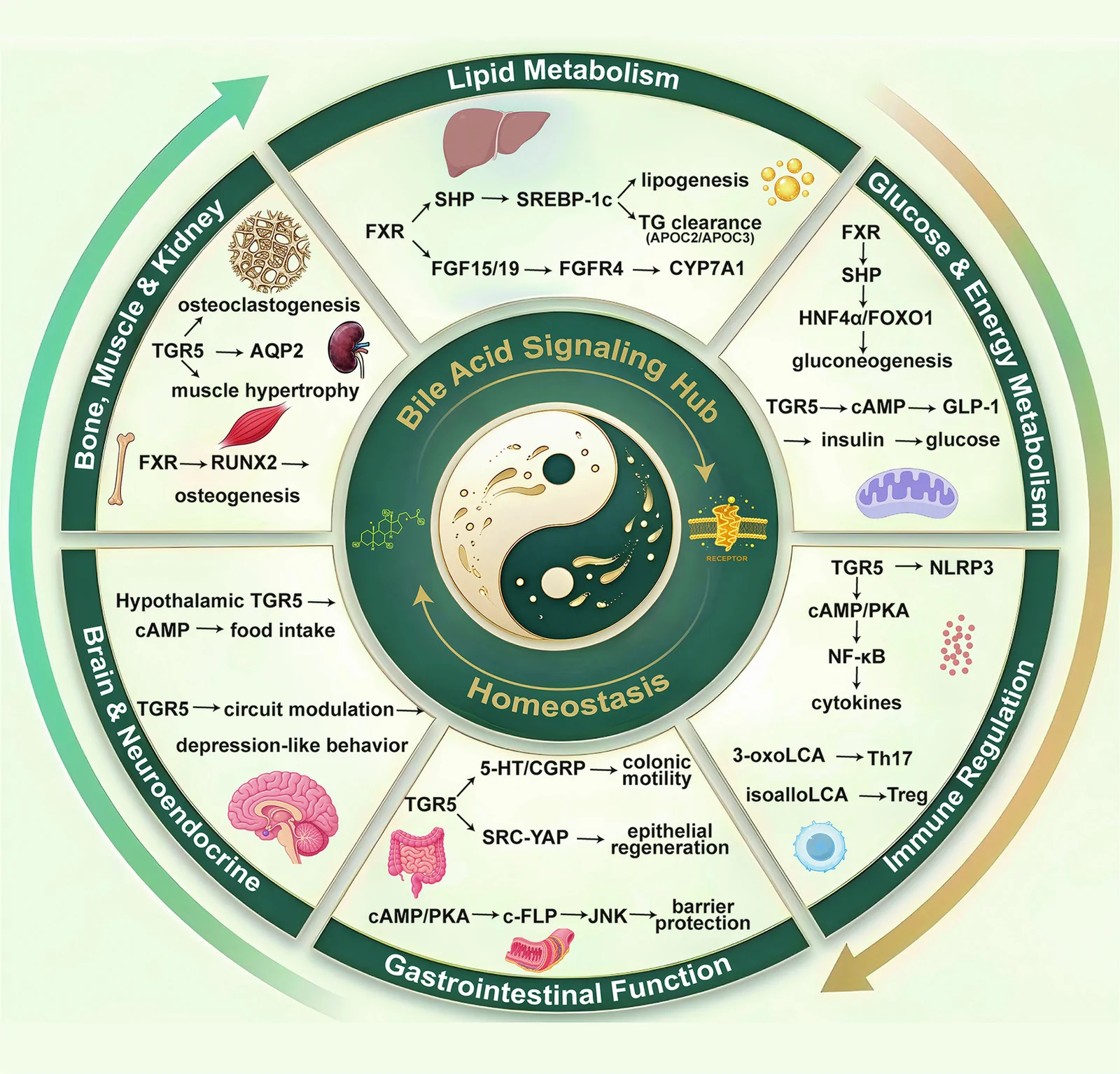
Physiological roles of BA signaling in systemic homeostasis. BAs function not only as digestive detergents but also as endocrine, paracrine, and local signaling molecules that coordinate whole-body physiology. Through nuclear receptors, particularly FXR, and membrane receptors such as TGR5, BA signals are shaped by ligand structure, local exposure, receptor distribution, and tissue-specific context. These pathways regulate lipid, glucose, and energy metabolism; maintain intestinal epithelial integrity and motility; shape immune and inflammatory responses; influence bone, muscle, and kidney physiology; and modulate neuroendocrine and brain-related functions. Thus, BA signaling forms a multi-organ communication network that links enterohepatic circulation to systemic metabolic, immune, and inflammatory homeostasis

Upstream control of BA synthesis, transport, enterohepatic circulation, and microbial remodeling shapes the intensity, duration, and receptor selectivity of BA signaling. By determining which BA ligands reach FXR, TGR5, and other receptors in the liver, intestine, and extrahepatic tissues, these processes coordinate lipid, glucose, energy, immune, and epithelial homeostasis (Fig. 5).

### Regulation of glucose, lipid, and energy metabolism

#### Lipid metabolism regulation

BAs are key regulators of lipid metabolism, primarily through activation of FXR. Upon BA binding, FXR forms a heterodimer with RXR and binds to FXR response elements, driving the expression of target genes that remodel hepatic lipid networks. The classic FXR–SHP–SREBP-1c axis exemplifies this: FXR induces SHP, which suppresses LXR-dependent SREBP-1c activity, thereby downregulating fatty acid synthesis genes (e.g., ACC, FASN, and SCD1) and reducing hepatic triglyceride accumulation. This pathway is a cornerstone of BA-mediated inhibition of lipid production [128].

Beyond direct inhibition, FXR activation targets lipid synthesis and re-esterification nodes (e.g., Scd1, Dgat2, and Lpin1) to selectively reduce hepatic monounsaturated and polyunsaturated fatty acids in mice and humans. It also modulates BA-derived intestinal signaling and lipid absorption, curbing nonalcoholic fatty liver disease (NAFLD) through effects on lipid synthesis, uptake, and remodeling [129]. Furthermore, under metabolic stress, FXR enhances fatty acid oxidation by remodeling transcriptional programs that promote mitochondrial adaptation and substrate utilization. Additionally, FXR regulates plasma triglycerides via lipoprotein metabolism by inducing APOC2 and suppressing APOC3 [130, 131].

Intestinal FXR induces FGF15/19 secretion in the ileum to aid systemic lipid homeostasis. FGF15/19 travels via the portal vein to hepatocytes, binds the FGFR4–β-Klotho complex, inhibits CYP7A1, and alters cholesterol and BA metabolism [37, 38, 132, 133]. This enterohepatic endocrine loop is further fine-tuned by an FGF15/19–TFEB–CYP7A1 regulatory circuit: reduced intestinal BA reabsorption weakens FGF15/19 signaling, activating transcription factor EB (TFEB) to promote cholesterol-to-BA conversion, thereby linking intestinal BA sensing to hepatic lipid processing [75]. However, drug development targeting the classic FXR–SHP–SREBP-1c axis and related pathways faces major challenges, such as off-target effects including cholestasis and dyslipidemia, which constitute a primary barrier to its clinical translation.

#### Glucose metabolism and energy metabolism regulation

BAs regulate glucose homeostasis via FXR and TGR5 pathways, forming a cross-organ network involving the liver, intestine, and pancreas. In the liver, FXR activation induces SHP expression, which suppresses gluconeogenic gene transcription by inhibiting HNF4α and FOXO1, thereby downregulating key enzymes such as PEPCK, G6Pase, and FBP1 and reducing hepatic glucose output—a core mechanism of BA-mediated gluconeogenesis inhibition [134]. Complementing this hepatic axis, intestinal FXR triggers PI3K-dependent FOXO1 phosphorylation via FGF15/19–FGFR4 signaling, further repressing gluconeogenic gene expression in hepatocytes and lowering fasting blood glucose to improve glycemic control [135]. In parallel, TGR5 mediates complementary metabolic benefits. In intestinal L cells, TGR5 activation by BAs stimulates glucagon-like peptide-1 (GLP-1) release, promoting insulin secretion, suppressing glucagon release, and delaying gastric emptying, thereby improving glucose tolerance and insulin sensitivity [136, 137]. Recent studies highlight that intestinal-targeted TGR5 activation offers a promising low-toxicity, sustained-efficacy strategy for enhancing glucose utilization [138].

Beyond glucose regulation, BAs regulate systemic energy balance through the TGR5–type 2 deiodinase (DIO2) axis. BA-activated TGR5 triggers cAMP signaling, upregulating DIO2 to enhance local thyroid hormone (T3) activation. This increases oxygen consumption and thermogenesis in brown adipose tissue and skeletal muscle, thereby counteracting diet-induced obesity and insulin resistance [139]. For instance, CDCA increases human brown adipose tissue activity and energy expenditure, whereas TGR5 deficiency impairs exercise-induced skeletal muscle adaptation [140, 141]. Together, these findings underscore the role of BAs in promoting metabolic flexibility and energy homeostasis via complementary FXR- and TGR5-dependent pathways.

### Intestinal homeostasis: barrier function, mucosal immunity, and microbial interactions

#### Innate and adaptive immune regulation

BAs significantly modulate gut innate immunity, mainly via FXR and TGR5 in monocytes/macrophages and dendritic cells (DCs) [142, 143]. In macrophages, FXR activation exerts anti-inflammatory effects by antagonizing NF-κB signaling, directly binding NLRP3 and caspase-1 to inhibit inflammasome assembly, and attenuating endotoxin-induced inflammatory responses [144]. TGR5 complements this by activating the cAMP–PKA pathway, which suppresses NF-κB nuclear translocation and blocks NLRP3-mediated IL-1β release [145, 146]. Beyond macrophages, TGR5 activation in DCs similarly dampens innate immune activation: secondary BAs such as DCA downregulate costimulatory molecules (CD40, CD80, CD86) and major histocompatibility complex II expression while reducing pro-inflammatory cytokines (IL-1β, IL-6, IL-12p70, TNF-α) [147]. This dampens Th1 cell responses and promotes intestinal immune tolerance.

Microbiota-derived secondary BAs further bridge innate and adaptive immunity by directly shaping T cell differentiation. The BA metabolite 3-oxoLCA inhibits Th17 cell differentiation through direct binding to RORγt, whereas isoalloLCA promotes regulatory T cell (Treg) generation [148, 149]. Indirectly, isoDCA reduces DC immunogenicity, enhancing peripheral Treg differentiation and reinforcing tolerance [150]. These mechanisms, encompassing direct T cell regulation by BA metabolites and antigen-presenting cell (APC)-mediated reprogramming, illustrate how microbiota-driven BA remodeling generates immunoactive signaling ligands that regulate the Th17/Treg balance and local inflammation. Notably, the hydrophobic–hydrophilic distribution of BA species shapes the relative abundance of these immunomodulatory ligands: hydrophobic secondary BAs (e.g., DCA and LCA) tend to promote pro-inflammatory Th17 responses, whereas hydrophilic BAs (e.g., UDCA) favor Treg induction and barrier protection, linking microbiota-dependent BA signaling to intestinal immune homeostasis. However, this oversimplified hydrophobicity–immunity dichotomy overlooks inter-individual gut microbiota heterogeneity and therefore cannot ensure consistent immunomodulatory outcomes across diverse human populations.

#### Gastrointestinal physiology and nutrient absorption

BAs serve as key luminal signals regulating gastrointestinal motility, epithelial barrier integrity, stem cell dynamics, and nutrient absorption via TGR5 and FXR. Regarding motility, BAs exert segment-specific effects. In the colon, TGR5 activation on enterochromaffin cells and enteric neurons stimulates 5-HT and calcitonin gene-related peptide (CGRP) release, driving peristaltic reflexes [151]. TGR5 disruption impairs these prokinetic responses, whereas microbiota-derived BA metabolites such as LCA enhance colonic transit [152]. Physiologically, BA levels maintain mucosal lubrication, but excessive local BA exposure triggers hypersecretion and diarrhea, underscoring the importance of ligand identity and tissue exposure in gut motility signaling.

Beyond motility, the BA–TGR5 axis critically governs intestinal stem cell (ISC) dynamics and barrier repair. TGR5 activation promotes crypt proliferation, lineage differentiation, and post-injury regeneration via the SRC–YAP pathway [153], while concurrently reducing epithelial apoptosis and tight junction disruption through cAMP/PKA/c-FLIP/JNK signaling in colitis models [154]. FXR contributes to barrier homeostasis in a context-dependent manner: it prevents dysbiosis and barrier dysfunction under steady state, yet its deletion paradoxically attenuates acute inflammatory injury. Conversely, pharmacological FXR activation mitigates cholestasis-related damage, likely via antimicrobial Reg3 programs [155], whereas the microbiota-derived metabolite 7-keto-LCA antagonizes FXR to sustain Wnt signaling and promote ISC repair—though excessive FXR inhibition may drive tumorigenesis under aberrant Wnt activation [156, 157]. Collectively, BAs control gut physiology through distinct, context-sensitive receptor pathways: TGR5 promotes motility and repair, whereas FXR fine-tunes barrier function, together linking ligand-specific BA signaling to intestinal integrity.

### Other physiological functions

Beyond the gut–liver axis, BAs act as systemic signaling molecules, influencing the brain, bone, muscle, and kidney through FXR, TGR5, and other receptors to coordinate whole-body homeostasis.

#### Brain function and neuroendocrine regulation

FXR and TGR5 are expressed in the hypothalamus and hippocampus, where BA–TGR5 signaling activates cAMP production to suppress appetite and improve energy metabolism—effects impaired in Tgr5-knockout mice, indicating direct central action rather than indirect gut-hormone mediation [32, 158]. TGR5 in lateral hypothalamic neurons also modulates depressive-like behaviors via glutamatergic signaling [159], suggesting that BA–TGR5 pathways influence neuronal excitability and plasticity, with potential as neuropsychiatric therapeutic targets [160].

#### Bone and skeletal muscle homeostasis

In bone, FXR promotes osteogenesis via ERK/β-catenin–RUNX2 signaling, while TGR5 suppresses osteoclastogenesis through AMPK; dual FXR/TGR5 agonism and the gut microbiota–BA–TGR5 axis further contribute to bone maintenance [161]. In skeletal muscle, exercise induces TGR5 via ATF6α-dependent UPR, supporting myocyte differentiation and hypertrophy [141]; muscle-specific TGR5 overexpression enhances glycolysis and glucose utilization, while the BA–TGR5–cAMP–DIO2–T3 axis increases mitochondrial oxygen consumption [139]. TGR5 also regulates fiber-type switching and regeneration via CaN and AKT/mTOR/FoxO3 pathways [162], collectively linking BA signaling to muscle metabolic flexibility and exercise adaptation.

#### Renal water-salt homeostasis

The kidney is a major BA target, with FXR and TGR5 highly expressed in renal tubules. FXR deletion impairs urine-concentrating ability and reduces AQP2 expression [163]. TGR5 activation in medullary collecting duct cells elevates cAMP, promoting AQP2/AQP3 transcription via PKA–CREB to increase urine osmolality—a mechanism paralleling the vasopressin–V2R–cAMP axis—thereby integrating BA signaling into renal water balance [164].

However, current knowledge of BA regulation of peripheral organs including the brain, bone, muscle and kidney consists mostly of isolated single-organ observations, and an integrated framework for circulating BA-mediated whole-body homeostasis via multi-organ crosstalk remains lacking.

## Dysregulation of BA signaling in disease

Disturbed BA signaling can arise from excessive or insufficient ligand exposure, disrupted enterohepatic feedback, altered microbial conversion, or accumulation of hydrophobic and cytotoxic BA species. These upstream changes reprogram receptor-dependent and membrane-mediated pathways, forming synergistic pathogenic mechanisms in liver, gastrointestinal, metabolic, and cardiovascular diseases.

### Liver diseases

#### Cholestatic liver disease

Cholestatic liver disease results from impaired bile secretion or flow, leading to pathological BA retention in the liver and circulation—characterized by altered signaling ligand exposure, particularly enrichment of hydrophobic conjugated species. Prolonged BA exposure damages hepatocytes and cholangiocytes, driving inflammation, fibrosis, and ultimately cirrhosis if unresolved [165, 166]. In response, elevated hepatic BAs activate FXR, which upregulates SHP and suppresses CYP7A1/CYP8B1 to reduce de novo synthesis, while intestinal FXR induces FGF15/19, which further inhibits hepatic CYP7A1 via FGFR4/β-Klotho signaling. FXR also promotes BA excretion by upregulating BSEP and other canalicular transporters. Concurrently, TGR5 in cholangiocytes enhances bile duct secretion and maintains biliary barrier integrity, while BAs suppress NLRP3 inflammasome activation via TGR5–cAMP–PKA signaling [146]. Consistently, Tgr5-deficient mice exhibit exacerbated liver injury under cholestatic conditions, underscoring TGR5-mediated protection in the biliary system. Despite the robust hepatoprotective effects of FXR and TGR5 demonstrated in preclinical cholestasis models, current targeted therapeutic strategies do not account for inter-patient heterogeneity in BA pool composition, a key underlying factor that explains the suboptimal response rates in several clinical trials.

#### MASLD/MASH

Metabolic dysfunction-associated steatotic liver disease (MASLD), formerly NAFLD, is the most prevalent chronic liver disease worldwide and may progress from simple steatosis/metabolic dysfunction-associated steatohepatitis (MASH) through fibrosis and cirrhosis to hepatocellular carcinoma (HCC) [167, 168]. BA dysregulation in MASLD/MASH involves altered ligand profiles, disrupted enterohepatic circulation, microbiota shifts, and impaired FXR–FGF19/TGR5 signaling, with abnormal plasma and fecal BA profiles particularly evident in advanced fibrosis [169]. Mechanistically, the FXR–FGF19–FGFR4 axis that normally suppresses BA synthesis via CYP7A1 inhibition is blunted in MASLD—despite preserved postprandial FGF19 secretion, hepatic FGFR4 signaling fails, leading to excessive BA production and sustained hepatic ligand exposure. Fibrosis progression also enriches plasma conjugated and secondary 12α-OH BAs, which are strongly linked to disease severity [169, 170]; these species promote hepatocyte injury via mitochondrial oxidative stress, ER stress, and cell death [171, 172]. Additionally, hydrophobic BAs exacerbate liver damage, while conjugated BAs disrupt PPARα signaling and promote lipid deposition, further aggravating steatotic injury [173]. Existing studies on hydrophobic BA-driven MASH liver injury mostly focus on direct hepatocyte cytotoxicity, while largely overlooking the uncharacterized multi-cell synergistic injury effect that activates intrahepatic non-parenchymal BA receptors to amplify inflammatory cascades via paracrine signaling.

#### Alcohol-associated liver disease

Alcohol-associated liver disease (ALD) is a leading cause of cirrhosis and HCC, driven by disrupted BA homeostasis. Patients with alcoholic hepatitis exhibit elevated plasma BAs, linked to disease severity [174], while alcoholic cirrhosis features increased fecal secondary BAs (DCA, LCA) due to intestinal inflammation, dysbiosis, and enhanced BA reabsorption [175]. Mechanistically, alcohol impairs BA transport—reducing hepatocyte uptake and increasing blood efflux [176]—while dysbiosis promotes primary-to-secondary BA conversion and accumulates FXR-antagonistic species, suppressing intestinal FXR–FGF19 signaling and paradoxically increasing de novo BA synthesis [177].

In ALD, altered BA ligand exposure and composition impair FXR and TGR5 signaling. FXR-deficient mice exhibit exacerbated liver injury after alcohol exposure [177, 178], and hepatic TGR5 activation normally suppresses NLRP3 inflammasome–mediated inflammation [179]. Systemically, elevated serum BAs activate TGR5 in brown adipose tissue to induce thermogenesis and fatty acid β-oxidation [180, 181]. Pharmacologically, the FXR agonist OCA, the TGR5 agonist INT-777, and the dual agonist INT-767 all attenuate liver injury in alcohol-exposed mouse models [179], yet these strategies overlook alcohol-induced intestinal barrier disruption that drives massive cytotoxic BA influx into the liver, which likely explains why such agents have failed to show positive outcomes in ALD clinical trials.

#### HCC

BAs promote HCC through systemic imbalances that remodel the tumor microenvironment. Central to this is the BA–FXR–YAP axis: FXR, a tumor-suppressive receptor, is downregulated in HCC, and its loss activates Wnt/β-catenin and YAP signaling, driving tumor growth—YAP further worsens BA retention, creating a vicious cycle, while YAP deletion blocks HCC progression [182, 183]. Consistently, reduced FXR expression in humans correlates with malignant HCC phenotypes, weakening BA homeostasis and anti-tumor effects [184]. Microbiota-derived DCA amplifies this axis via enterohepatic circulation, inducing DNA damage, stellate cell senescence, and inflammation [185, 186]. TGR5 plays context-dependent roles: high GPBAR1 expression is linked to poor prognosis, suggesting pro-tumorigenic functions [187]. Beyond direct signaling, BA composition shapes hepatic anti-tumor immunity—primary BAs promote CXCL16-mediated recruitment of CXCR6^+^ NKT cells, whereas microbiota-derived secondary BAs suppress this axis, facilitating immune escape [188]. Elevating primary BAs reshapes the immune microenvironment via the microbiota–butyrate–CXCL14 axis, restoring anti-tumor immunity [189]. The established paradigm defining the BA–FXR–YAP axis does not account for BA profile heterogeneity across etiologically distinct HCCs, such as distinct BA profiles between ALD- and MASLD-associated HCC, so single-targeted BA receptor interventions cannot achieve uniform anti-tumor efficacy across all HCC subtypes.

### Gastrointestinal disorders

#### Inflammatory bowel disease

Inflammatory bowel disease (IBD), including ulcerative colitis (UC) and Crohn's disease (CD), results from genetic, environmental, microbial, and immune factors, with disrupted BA homeostasis serving as a key amplifying factor [14, 190–192]. BA dysregulation in IBD operates at multiple levels. In ileal CD, BA malabsorption occurs as a result of reduced ASBT/SLC10A2 expression and persists even during remission in both UC and CD [193]. This malabsorption drives secretory diarrhea and correlates with disease severity [194]. Concurrently, IBD-associated dysbiosis shifts the BA pool toward increased primary BAs and decreased anti-inflammatory secondary species (e.g., DCA), exacerbating mucosal inflammation particularly under high-fat diets [195]. These compositional changes impair FXR–FGF19 signaling, weakening feedback inhibition of BA synthesis and compromising intestinal barrier integrity [196]. TGR5 is upregulated in inflamed CD tissues as a compensatory response, and its deficiency increases colitis susceptibility; however, secondary BA depletion limits TGR5-mediated protection [197]. Beyond FXR and TGR5, reduced epithelial VDR expression impairs barrier defense in CD/UC, while impaired PXR signaling fails to antagonize NF-κB–driven inflammation, further aggravating mucosal damage [198]. Collectively, these BA-dependent mechanisms suggest a pivotal role for BA dysregulation in IBD pathogenesis; however, whether it acts as a primary driver or a secondary consequence of inflammation remains an unresolved question critical for targeted therapy development.

#### Bile acid diarrhea

Bile acid diarrhea (BAD) accounts for ~30% of chronic watery diarrhea in diarrhea-predominant irritable bowel syndrome (IBS-D) and functional diarrhea, and is classified into three types: Type I (ileal damage-induced malabsorption), Type II (primary, with impaired BA synthesis feedback), and Type III (secondary to cholecystectomy or infections) [199].

The main pathological mechanism of BAD is insufficient negative feedback via the ileal FXR–FGF19–hepatic FGFR4/β-Klotho axis [200]. BAD patients show reduced fasting serum FGF19 correlating with selenium homocholic acid taurine (SeHCAT) retention and inversely with serum C4 [201, 202]; even with normal ileal histology, distal ileal FGF19 mRNA and protein are diminished, indicating impaired BA sensing and endocrine function [200]. Consequently, elevated C4 reflects increased hepatic BA synthesis, while altered BA profiles reveal higher unconjugated and primary species in serum and feces [203]. In IBS-D subgroups with BA excess, Clostridia-rich microbiota elevates UDCA levels, further impairing FXR–FGF19 signaling and hepatic feedback [204].

These BA imbalances drive BAD symptoms through three parallel mechanisms. First, DCA/LCA activate TGR5 on enteroendocrine and sensory neurons, enhancing colonic motility via 5-HT/CGRP release—an effect absent in Tgr5-knockout mice [151]. Second, excessive BAs stimulate colonic Cl⁻ secretion; FXR agonists suppress this response, and LCA inhibits secretion via FXR-mediated cystic fibrosis transmembrane conductance regulator (CFTR) downregulation [205]. Third, membrane-active BAs (especially DCA) disrupt tight junction proteins and increase epithelial permeability, contributing to urgency and incontinence [206, 207]. Collectively, while these three parallel BA-driven pathways explain the core symptomatic manifestations of BAD, current clinical diagnostic workflows still cannot stratify patients based on which of these three mechanisms dominates, which limits the precision of BA-targeted therapeutic regimens for BAD.

#### Colorectal cancer

Colorectal cancer (CRC) is the third most prevalent malignancy worldwide, with high-fat diets, obesity, and altered intestinal microbiota–BA metabolism serving as key drivers beyond genetic factors [208]. High-risk individuals and CRC patients exhibit elevated fecal primary (CA, CDCA) and secondary BAs (DCA, UDCA) [209]. Mechanistically, BA profile shifts—particularly DCA enrichment—promote CRC through multiple parallel routes. At the genomic level, DCA induces DNA damage, disrupts p53-mediated repair, and drives genomic instability [210]. At the immune level, elevated DCA inhibits Ca^2^⁺–NFAT2 signaling in CD8⁺ T cells, impairing anti-tumor immunity and facilitating tumor growth [200].

Receptor signaling is equally critical. The protective FXR axis is frequently suppressed: FXR downregulation promotes proliferation and metastasis via DNA methylation and KRAS signaling, while its overexpression inhibits tumor growth; high-fat diets and APC inactivation further block intestinal FXR through DCA-mediated antagonism, driving Lgr5⁺ stem cell expansion and adenoma progression [211]. TGR5/GPBAR1 plays context-dependent roles—reduced expression correlates with poor prognosis, whereas UDCA suppresses proliferation via TGR5 activation [212, 213]. Beyond receptors, non-canonical pathways amplify BA-driven oncogenesis: DCA triggers EGFR–MAPK activation [121, 214]; LCA induces IL-8 via ROS–NF-κB/ERK/AP-1 and enhances invasiveness [215, 216]; CA upregulates matrix metalloproteinase-9 (MMP-9) through ROS–MAPK–NF-κB/AP-1 signaling [217]. While these multi-layered oncogenic mechanisms establish BA dysregulation as a central hub linking diet, microbiota, and CRC pathogenesis, current research lacks a clear causal threshold for BA concentration and compositional shift that triggers inflammation-to-malignancy transition.

### Metabolic and systemic diseases

#### Obesity and metabolic syndrome

Metabolic syndrome, affecting 28.4% globally in 2023, increases risks of diabetes and cardiovascular disease, partly through dysregulated BA signaling [218]. Intestinal FXR plays a context-dependent role. On the one hand, intestine-restricted FXR agonism (e.g., fexaramine) boosts ileal FGF15, remodels BA composition, and ameliorates insulin resistance—FXR activation also enhances TGR5–GLP-1 signaling via microbiota shifts, aiding glucose homeostasis [219]. On the other hand, intestine-selective FXR inhibition with Gly-MCA ameliorates obesity, insulin resistance, and hepatic steatosis by suppressing intestinal FXR-dependent ceramide synthesis and counteracting microbiota-driven pro-obesity FXR signaling [220–222]. These opposing outcomes highlight that intestinal FXR activation and inhibition exert distinct systemic effects depending on metabolic context.

TGR5 generally exerts protective effects across multiple tissues: it stimulates GLP-1 release, increases energy expenditure, and maintains glucose homeostasis, with human studies confirming metabolic benefits of BA-based therapies [136, 140, 223]. Beyond metabolism, TGR5 signaling modulates immune–metabolic crosstalk by suppressing chemokine expression (CCL2, CCL3, CCL4), limiting macrophage infiltration, and attenuating diet-induced inflammation [224]. Emerging evidence further implicates TGR5 in neuro–immune–metabolic interactions within adipose tissue, suggesting broader systemic roles in energy balance [225].

#### Type 2 diabetes mellitus

Type 2 diabetes mellitus (T2DM) is a chronic metabolic disorder characterized by insulin resistance, β-cell dysfunction, and chronic hyperglycemia, often accompanied by obesity and cardiovascular complications [226]. T2DM patients, particularly those with high homeostatic model assessment of insulin resistance (HOMA-IR), exhibit altered plasma and fecal BA profiles with notable shifts in BA proportions despite stable or increased total plasma BA levels [227].

Mechanistically, insulin resistance in T2DM is linked to increased BA synthesis and impaired enterohepatic transport. Postprandial BA levels rise more prominently in T2DM patients, driven by elevated DCA and altered CA/UDCA levels [228]. This reflects impaired FXR feedback: despite higher circulating FXR-agonistic BAs, the BA–FXR–FGF19 axis fails to compensate due to low or unresponsive FGF19. Consistently, intestinal biopsies from T2DM patients show reduced ASBT, OSTα/β, FXR, and FGF19 mRNA expression [229]. In contrast, BA-mediated TGR5 activation exerts protective metabolic effects—stimulating GLP-1 and peptide YY (PYY) release via Gs–cAMP–PKA/EPAC signaling, enhancing insulin secretion and postprandial glycemic control. Targeted ileal or colonic delivery of conjugated BAs further improves glucose regulation by activating TGR5-expressing L cells and directly boosting β-cell insulin secretion [230, 231]. Thus, the imbalanced FXR–TGR5 axis in T2DM, with impaired FXR feedback and compensatory TGR5-mediated GLP-1/PYY release, drives the BA-related metabolic phenotype.

#### Atherosclerosis

Atherosclerosis (AS) is a chronic inflammatory arterial disease marked by lipid accumulation, endothelial dysfunction, and immune cell infiltration. BA receptors exhibit tissue-specific and sometimes opposing roles in AS pathogenesis.

FXR signaling shows context-dependent effects. While hepatic FXR generally promotes cholesterol clearance, intestinal FXR paradoxically exacerbates AS under high-cholesterol conditions—intestinal FXR-deficient ApoE⁻/⁻ mice exhibit reduced plaque formation [232]. Excessive intestinal FXR activation suppresses hepatic BA synthesis and induces inflammation via the SMPD3–ceramide pathway, with elevated serum FGF19 in hypercholesterolemic patients further supporting its pro-atherogenic role [233]. TGR5, in contrast, is broadly atheroprotective: it inhibits macrophage inflammation and oxidized low-density lipoprotein (LDL) uptake, reducing plaque macrophage burden [234]; endothelial TGR5 activation enhances eNOS-mediated NO production, suppresses vascular cell adhesion molecule-1 (VCAM-1), and inhibits monocyte adhesion [235]; and microbiota-derived BAs alleviate coronary artery disease via platelet TGR5 by inhibiting thrombosis [5].

Notably, FXR and TGR5 act synergistically in AS. The dual agonist INT-767 reduces serum cholesterol, aortic plaque, and inflammation in ApoE⁻/⁻ and LDLR⁻/⁻ mice by combining TGR5-mediated anti-inflammatory signaling (PKA–NF-κB in macrophages) with FXR-driven lipid lowering and BA reprogramming [236]. Dual receptor inactivation abolishes INT-767's benefits and worsens aortic inflammation [237], underscoring that FXR and TGR5 cooperatively regulate AS—FXR governs systemic lipid metabolism, whereas TGR5 targets immune and endothelial functions—positioning combined BA receptor activation as a promising therapeutic strategy. Beyond BA receptor signaling, BA transporters also contribute to AS. For instance, Mdr2/ABCB4, a key transporter in BA homeostasis, has been linked to cardiovascular diseases through indirect mechanisms. Bone marrow-derived ABCB4 deficiency aggravated atherosclerotic lesion development in LDL receptor–deficient mice, probably by promoting macrophage foam-cell formation [238]. In humans, ABCB4 dysfunction is primarily linked to cholestatic liver diseases and altered BA profiles; therefore, its contribution to AS through disrupted BA balance, lipid metabolism, and inflammation remains mechanistically plausible but clinically underexplored [239, 240].

### Biliary tract diseases

#### Primary biliary cholangitis

Primary biliary cholangitis (PBC) is a chronic liver disease marked by immune-mediated bile duct damage, causing BA imbalance, epithelial dysfunction, and immune-metabolic disruption [241]. FXR activation reduces hepatic BA synthesis and uptake while promoting efflux via transporters (e.g., OSTα/OSTβ), alleviating toxicity [242]. However, in PBC, uptake transporters decline while efflux transporters remain stable or increase [243, 244]. Clinically, FXR agonists (e.g., OCA) lower BA synthesis and improve biochemical markers (e.g., FGF19) in UDCA-refractory patients but fail to halt immune-mediated ductal destruction or improve long-term outcomes, leading to EU (2024) and U.S. (2025) regulatory withdrawals. Thus, FXR-targeted therapy in PBC is fundamentally limited by its inability to address the autoimmune component.

TGR5 signaling is also compromised in PBC despite stable receptor expression in bile duct cells. Cholestasis depletes secondary BAs, limiting TGR5 activation and weakening bile duct defense, which increases intestinal permeability and systemic inflammation. A central mechanism involves miR-506-mediated anion exchanger 2 (AE2) downregulation, disrupting the bicarbonate umbrella via the secretin–cAMP–CFTR axis and amplifying BA toxicity and immune activation [245, 246]. AE2 deficiency further impairs autophagy, exposing PDC-E2 (a major autoantigen) and promoting cellular senescence, thereby hindering tissue repair [247]. In advanced PBC, the secretin/secretin receptor axis declines; however, exogenous secretin restores biliary secretion in preclinical models, reducing ductular reactions, immune infiltration, and fibrosis [248]. Collectively, BA receptor dysfunction in PBC reflects a dual failure: FXR agonism cannot reverse autoimmunity, while impaired TGR5 signaling and AE2 deficiency compromise epithelial protection and immune homeostasis, underscoring the need for therapies that simultaneously protect bile ducts and rebalance immune–metabolic responses to slow disease progression.

#### Primary sclerosing cholangitis

Primary sclerosing cholangitis (PSC) is a chronic cholestatic liver disease characterized by intra- and extrahepatic bile duct inflammation and strictures, frequently associated with IBD. Pathogenesis involves immune dysregulation, genetic susceptibility, and gut–liver axis dysfunction, with BA homeostasis imbalance as a central driver [249, 250]. PSC patients exhibit adaptive intestinal changes including upregulated FXR protein, reduced ASBT, and increased OSTβ, reflecting altered BA sensing and efflux [250]. Although baseline FGF19 is normal, CDCA induces prolonged FGF19 elevation with sustained BA accumulation; in poor-prognosis groups, low baseline C4 fails to be suppressed despite FGF19 rise, indicating impaired BA feedback [251]. Low C4 correlates with shorter survival, suggesting FXR agonism is ineffective in advanced PSC [252]. This aligns with the PRIMIS trial, where cilofexor failed to slow fibrosis despite pharmacodynamic effects [253].

TGR5 signaling is also diminished in PSC, unlike its relatively preserved role in PBC. TGR5 is downregulated in PSC patients and Abcb4⁻/⁻ mice; its deletion exacerbates ductal injury while restoration attenuates inflammation [254]. norUDCA partially restores TGR5 expression, supporting its protective role [254]. TGR5 downregulation impairs epithelial defenses and promotes BA-driven ductal injury and fibrosis, highlighting the need for therapies that restore TGR5 signaling to protect bile ducts and rebalance immune–metabolic responses [166, 255]. Both FXR and TGR5 are dysregulated in PSC: FXR agonism fails in advanced disease while TGR5 loss impairs epithelial protection, indicating the need for therapies that simultaneously restore BA homeostasis and bile duct integrity. This "double failure" of FXR and TGR5 signaling directly explains why single-target therapies have shown limited efficacy in PSC, underscoring the urgent need for combination strategies that restore both signaling arms.

#### Cholangiocarcinoma

CCA arises from biliary epithelial cells, with outcomes varying by anatomical location and molecular subtype. Multi-omics analyses reveal elevated conjugated BAs, downregulated metabolic enzymes, and intratumoral BA accumulation, all linked to poor prognosis and immunosuppression [256–258].

BA receptors exert context-dependent and often opposing roles in CCA. FXR is generally downregulated in CCA tissues; its agonists (e.g., OCA) inhibit proliferation, migration, and invasion via IL-6/epithelial–mesenchymal transition (EMT) suppression in intrahepatic CCA, supporting a tumor-suppressive function [259]. TGR5, in contrast, is consistently upregulated and pro-tumorigenic. TGR5 activation promotes cholangiocyte proliferation via EGFR/ERK signaling and inhibits apoptosis [102], while TCA/GCA drive S1PR2-dependent ERK1/2, AKT, NF-κB, and COX-2/PGE2 cascades that enhance invasion and 3D ductal expansion [108, 260, 261]. Beyond tumor cells, excessive BAs activate TGR5 on cancer-associated fibroblasts, inducing CXCL10-mediated recruitment of immunosuppressive neutrophils and driving EMT/metastasis; blocking GPBAR1 or CXCL10 restores pembrolizumab sensitivity [258]. Thus, TGR5 bridges BA dysregulation to CCA progression and immune evasion, while FXR activation remains protective, supporting combined TGR5 inhibition and FXR agonism as a therapeutic strategy.

## Therapeutic targeting of BA signaling

### BA receptor-pathway modulation

In the BA signaling network, receptors serve as central hubs that translate ligand structure, local exposure, and tissue context into biological outputs. Structurally distinct BAs selectively engage nuclear or membrane receptors to activate transcriptional programs or rapid signaling cascades, converting upstream BA generation and distribution into biological effects. Therapeutic strategies, therefore, aim to restore homeostasis by selectively modulating receptor signals and correcting pathological BA exposure. Current drug development focuses on FXR, TGR5, and the downstream FGF19 axis (Table 2).

**Table 2:** Therapeutic agents targeting FXR, TGR5, and FGF19 pathways in liver diseases

Therapeutic target	Representative drug	Drug characteristics	Mechanism of action	Indication/disease	Animal studies	Clinical application/clinical trials	References
FXR agonists	OCA	Semi-synthetic BA derivative; potent FXR agonist	Potently activates FXR; induces hepatic SHP to suppress CYP7A1/CYP8B1, and reduces de novo BA synthesis. It also upregulates BSEP and OSTα/β to promote BA efflux and induces ileal FGF19 signaling, thereby inhibiting hepatic BA synthesis and attenuating lipogenesis, inflammation, and fibrosis	PBC	In LCA-induced cholestatic mice, OCA improved cholestasis and hepatocyte apoptosis, reduced the toxic BA burden, and upregulated transporters including BSEP, MRP2, and MRP3	OCA was the first FXR agonist approved for clinical use; however, postmarketing studies failed to demonstrate clinical benefit, and an increased risk of liver transplantation or death was observed in early-stage PBC	[262, 263]
				MASH/NASH	In AMLN diet-fed ob/ob mice, OCA reduced hepatic lipid accumulation, inflammation, and fibrosis, improving NASH histologic features	Showed some effect on improving hepatic fibrosis, but further indication development was constrained by the efficacy-safety balance	[264]
	Tropifexor (LJN452)	Highly selective non-bile-acid FXR agonist	Enhances transcription of FXR target genes, suppresses BA synthesis and hepatic lipid accumulation, and has anti-inflammatory and anti-fibrotic effects	MASH/NASH	In AMLN and STAM rodent NASH models, LJN452 significantly reduced steatosis, inflammation, and fibrosis, with stronger efficacy than OCA at low doses	FLIGHT-FXR phase IIa/b showed reductions in ALT and MRI-PDFF, but histologic endpoints were not sufficiently robust; not marketed	[265, 266]
				PBC	In a bile duct ligation (BDL) cholestatic juvenile pig model, LJN452 attenuated liver injury, ductular reaction, and fibrosis, and activated FGF19–SHP/suppressed CYP7A1; subsequent studies further suggested improvement of metabolic disturbances associated with hyperammonemia	Phase II clinical development has been conducted, but clinical use remains limited	[267, 268]
	Cilofexor (GS-9674)	Nonsteroidal, intestine-biased FXR agonist	Suppresses de novo BA synthesis and improves cholestatic and inflammatory signaling	PSC	In an Mdr2-/- sclerosing cholangitis model, GS-9674 improved AST/ALP and the BA profile, reduced fibrosis, and increased bile flow/biliary HCO_3_^−^ output	Early PSC trials improved biomarkers such as ALP and C4; the phase III PRIMIS trial failed to significantly reduce fibrosis progression in non-cirrhotic PSC	[253, 269, 270]
				NASH	In a Wistar rat NASH model, GS-9674 reduced portal pressure and decreased hepatic fibrosis/hepatic stellate-cell activation, showing both anti-fibrotic and hemodynamic benefits	In ATLAS, its use in NASH combination therapy also failed to meet the primary endpoint	[271, 272]
	Nidufexor (LMB763)	Partial FXR agonist/modulator	Aims to improve the tolerability window through partial agonism	MASH/NASH	In mouse NASH models, LMB763 produced FXR-dependent in vivo gene regulation and reduced steatosis, inflammation, and fibrosis	Has entered phase II clinical studies	[273, 274]
	EDP-305	Highly selective FXR agonist	Enhances expression of FXR target genes, reduces BA burden, and inhibits fibrosis	MASH/NASH	In MCD diet-induced steatohepatitis mice, EDP-305 significantly suppressed liver injury and fibrosis, and showed anti-fibrotic features without worsening the ductular reaction	ARGON-1 phase II showed improvements in ALT and MRI-PDFF	[275, 276]
	MET409	Structurally optimized FXR agonist	Preserves fat-lowering/anti-fibrotic signaling while reducing pruritus and LDL-C elevation	NASH/MASH	In biopsy-confirmed fibrotic DIO-NASH mice, MET409 improved the NAS score and exerted anti-fibrotic effects while improving metabolic and biochemical parameters	A phase Ib trial showed reduced liver fat within 12 weeks; it has not reached the marketing stage	[277, 278]
TGR5 agonists	INT-777	Selective TGR5 agonist	Activates Gs–cAMP–PKA signaling, promotes GLP-1 secretion from intestinal L cells, and improves insulin secretion and sensitivity; inhibits NF-κB inflammatory signaling in macrophages; promotes energy expenditure and mitochondrial oxidative metabolism	Type 2 diabetes	In db/db mice, INT-777 promoted α-cell PC1 expression and islet GLP-1 release, increased β-cell proliferation/insulin synthesis, and improved glucose tolerance and insulin sensitivity	/	[279]
				Obesity/metabolic syndrome	In obese mice, INT-777 activated TGR5 to promote GLP-1 secretion from intestinal L cells, improving glucose tolerance and enhancing pancreatic islet and hepatic metabolic function	/	[136]
	INT-767	Dual FXR/TGR5 agonist	Suppresses BA synthesis and promotes bicarbonate secretion; provides anti-inflammatory/anti-fibrotic effects and can remodel BA composition and hydrophobicity	PSC	In an Mdr2-/- cholangiopathy model, INT-767 reduced inflammation and biliary fibrosis and promoted secretion of HCO_3_^−^ enriched bile	/	[280]
				MASH/NASH	In ob/ob-AMLN and WD-NASH models, INT-767 improved steatosis, inflammation, and collagen deposition	/	[281, 282]
	SB-756050	Selective TGR5 agonist	Promotes cAMP generation and GLP-1 release	Type 2 diabetes	/	The first human TGR5 clinical candidate; pharmacodynamic responses were highly variable, no clear glucose-lowering benefit was established, and subsequent development stalled	[103]
	RDX8940	Intestine-restricted TGR5 agonist	Retains incretin effects while reducing systemic/gallbladder-related adverse effects	Type 2 diabetes	In Western diet-induced insulin-resistant mice, RDX8940 promoted gut-derived GLP-1/GLP-2/PYY secretion, improved glycemic control and insulin sensitivity, and did not inhibit gallbladder emptying	Still in early translational/clinical development; no established role as standard therapy	[283]
	BAR501	Semi-synthetic TGR5 agonist	Has potential anti-inflammatory, anti-fibrotic, and vascular/biliary protective effects	NASH; portal hypertension; cholangiopathy models (preclinical)	In HFD-F mice, BAR501 reversed steatohepatitis and vascular injury; in portal hypertension models, it reduced portal pressure and improved endothelial function; Abcb4-/- studies suggest that it can attenuate biliary inflammation and hepatic fibrosis	Preclinical stage; more suitable as evidence for target druggability than as a near-term clinical candidate	[284–286]
FGF19 analogs	NGM282 (Aldafermin)	Engineered FGF19 analog	Binds FGFR4/β-Klotho, potently suppresses CYP7A1 and reduces de novo BA synthesis; improves lipid metabolism and attenuates hepatic steatosis, inflammation, and fibrosis; designed to avoid the STAT3-mediated pro-tumorigenic signaling of native FGF19	MASH/NASH	Its engineered FGF19 analog M70 rapidly lowered ALT/AST and improved steatosis, inflammation, and fibrosis in HFFCD mice	A randomized phase II trial confirmed rapid reduction of liver fat content; ALPINE II/Ⅲ did not meet the primary histologic endpoint, although multiple secondary endpoints improved	[287–289]
				PSC	In an Mdr2-/- cholangiopathy model, M70 rapidly reversed liver injury/inflammation, attenuated biliary fibrosis, and reduced the BA pool	ALPINE-PSC (Phase IIb/Ⅲ) aimed to evaluate the anti-fibrotic effect of aldafermin in PSC using ELF score as the primary endpoint, but the trial was terminated early because the design had to be adjusted to meet regulatory requirements, and no results are available; randomized controlled trials showed reductions in C4 and some inflammatory/fibrosis markers, but improvements in key cholestatic endpoints such as ALP were limited; clinical benefit remains uncertain and no treatment standard has been established	[290–292]

#### FXR agonists

The prototypical FXR agonist, OCA, initially showed promise in cholestatic diseases by reducing toxic BA burden and improving bile flow [262]. However, subsequent trials failed to confirm clinical benefit and raised safety concerns, prompting the FDA to decline approval of OCA for nonalcoholic steatohepatitis (NASH) in 2023. This spurred development of next-generation FXR agonists with three defining improvements. First, tissue-selective agonists (e.g., tropifexor and cilofexor) avoid the hydrophobic toxicity of natural BAs, minimizing off-target damage and enhancing safety [293]. Second, intestine-restricted agonists reduce intrahepatic side effects while preserving extrahepatic FGF19 induction, protecting liver function. Third, partial agonists (e.g., nidufexor) widen the therapeutic window by moderating receptor activation, improving tolerability and adherence [273, 274]. Despite these advances, histological endpoints remain inconsistent, particularly in MASH/NASH, where FXR modulation alone cannot fully correct BA dysregulation. Thus, FXR agonism must be complemented by targeting parallel pathways such as TGR5 for comprehensive metabolic and anti-inflammatory restoration. The marked therapeutic heterogeneity across all FXR-targeted clinical trials has never been systematically analyzed retrospectively using the baseline BA ligand stratification framework proposed in this review, which likely explains why numerous late-stage trial failures are misattributed solely to insufficient drug efficacy.

#### TGR5 agonists

TGR5 serves as a key target for BA signaling, incretin effects, and immune regulation, with its activation exerting therapeutic effects through dual mechanisms: at the metabolic level, selective TGR5 agonists (e.g., INT-777) significantly improve pancreatic islet function, insulin sensitivity, and glucose tolerance in obese and diabetic animal models; at the biliary-inflammatory level, FXR/TGR5 dual agonists (e.g., INT-767) reduce biliary inflammation, hepatic steatosis, and fibrosis while promoting bicarbonate-rich bile secretion in models of PSC and MASH/NASH [281, 282]. However, the first human TGR5 candidate drug, SB-756050, demonstrated unstable glycemic control and variable pharmacodynamic responses in patients with type 2 diabetes [103], while systemic TGR5 activation may trigger side effects such as gallbladder filling. Current research focuses on developing gut-restricted agonists (e.g., RDX8940) to preserve incretin effects while minimizing systemic exposure and gallbladder risks [283]. Despite limited monotherapy clinical evidence, the potential of TGR5 agonists to improve metabolism and inflammation positions them as promising core components for combination therapies aimed at restoring BA signaling homeostasis alongside FXR modulators, FGF19 analogs, or microbiome interventions.

#### FGF19 analogs

FGF19 analogs mimic the ileal FXR–FGF19 axis to suppress CYP7A1, reducing BA synthesis and toxic hepatic exposure. The engineered analog NGM282 (Aldafermin) retains BA synthesis inhibition and metabolic benefits (e.g., reduced steatosis and improved insulin sensitivity) while minimizing the pro-tumorigenic risks of native FGF19. In NASH, Aldafermin rapidly improves steatosis and markers of injury, inflammation, and fibrosis, with biopsy-confirmed short-term histological gains [287, 290]; however, the ALPINE II/Ⅲ trials failed to reverse long-term fibrosis, indicating that BA synthesis suppression alone is insufficient for multifactorial MASH/NASH. In PSC, Aldafermin reduces BA synthesis markers (e.g., C4) and modulates inflammatory-fibrotic pathways but shows limited effect on cholestatic markers (e.g., alkaline phosphatase [ALP]), leaving clinical benefit uncertain [288, 291]. Collectively, FGF19 analogs effectively reduce toxic BA exposure at the source but do not fully correct microbiome-driven secondary BA signaling or receptor-context imbalance. Thus, combining FGF19 analogs with FXR/TGR5 modulators or microbiome-targeted strategies is needed to coordinate BA synthesis control, ligand remodeling, and tissue-specific signaling for sustained remission.

### Modulating BA signal exposure through transporters and synthetic enzymes

Emerging strategies increasingly emphasize precise regulation of BA signal exposure, enterohepatic distribution, and ligand composition. Targeting key transporters (e.g., ASBT and NTCP) or synthetic enzymes (e.g., CYP8B1) reshapes receptor ligand availability and local BA signaling, linking homeostasis to cholestatic control, metabolic improvement, and gastrointestinal function.

#### Inhibition of ileal BA reabsorption

ASBT (SLC10A2) mediates ~95% of intestinal BA reabsorption and is a pivotal node for ileal BA sensing and enterohepatic signal strength. ASBT inhibitors (e.g., odevixibat, maralixibat, and elobixibat) block terminal ileal recycling, simultaneously increasing fecal BA excretion and remodeling hepatic BA composition. Preclinically, the non-absorbable ASBT inhibitor SC-435, combined with Asbt knockout, quadruples fecal BA excretion in high-fat diet-fed mice, abolishes ileal FGF15, shifts hepatic BA composition toward hydrophilicity, and improves steatosis, cholesterol accumulation, and glucose tolerance [294]. Clinically, odevixibat significantly improved pruritus in pediatric PFIC (53.5% vs 28.7% placebo) and reduced serum BAs (33.3% vs 0%) [295]; maralixibat sustained pruritus relief in Alagille syndrome with rapid relapse upon withdrawal, confirming a reversible mechanism [296, 297]; linerixibat produced significant 24-week pruritus improvement in PBC-related cholestasis, though diarrhea was frequent (61% vs 18%) [298]. Notably, elobixibat exploits the same mechanistic principle of increased colonic BA exposure to promote secretion and motility in chronic constipation, increasing bowel movement frequency with rapid onset [299]. Collectively, while ASBT inhibition couples reduced enterohepatic recycling with ligand remodeling to treat cholestasis, metabolic dysfunction and motility disorders, the lack of patient stratification criteria makes it impossible to predict treatment response and the risk of severe diarrhea.

#### Hepatocyte uptake and distribution remodeling

NTCP inhibition (e.g., Myrcludex B/Bulevirtide) represents a paradigm shift from traditional BA-lowering strategies: rather than reducing the total BA pool size, it blocks hepatic uptake of conjugated BAs, redistributing them to the portal vein and systemic circulation. In healthy volunteers, a single Myrcludex B dose increased total plasma BA exposure ~19.2-fold, with individual conjugated species (e.g., TCA) rising > 100-fold, yet without cholestatic manifestations [300], suggesting reduced intracellular hepatic burden despite systemic elevation. Preclinically, NTCP inhibition lowered hepatic BA load and improved bile phospholipid/BA ratios in DDC, BDL, and Atp8b1⁻/⁻ models; however, in Abcb4⁻/⁻ mice lacking biliary phospholipid secretion, the same inhibition worsened injury, highlighting that protection depends on intact bile buffering capacity [301].

Beyond cholestasis, NTCP inhibition confers metabolic benefits. Prolonged systemic BA exposure stimulated TGR5-dependent GLP-1 secretion, reducing body weight and steatosis in obese mice [302], while Bulevirtide lowered cholesterol and attenuated AS in humanized BA uptake models [303]. Clinically, Bulevirtide produces marked serum BA elevation, yet pruritus remains mild and transient with no clear BA-level correlation [304, 305], suggesting surprising tolerance to systemic BA pool expansion. Nevertheless, long-term systemic BA accumulation raises concerns about occult cardiovascular or neuroendocrine toxicity, and whether chronic BA redistribution alters the circulating ligand spectrum remains unexplored. Virtually all completed NTCP inhibitor clinical trials have only monitored total serum BA levels, without stratifying analysis by circulating BA ligand composition and tissue-specific signaling outputs, leaving potential long-term cardiovascular and neuroendocrine toxicity undetectable at early stages. Future studies must prioritize compositional profiling of circulating BA ligands under NTCP inhibition to comprehensively assess long-term safety and tissue-specific signaling consequences.

#### Enzyme-targeted composition remodeling

BA structural properties are shaped by enzymatic branch-point control, with CYP8B1 positioned at the classical pathway branch point as a central regulatory node. By controlling the CA/CDCA ratio, CYP8B1 directly shapes BA micellar properties, hydrophobicity, intestinal lipid absorption, and receptor ligand environments. Clinically, elevated 12α-hydroxylated BAs (e.g., CA) correlate with insulin resistance and dyslipidemia, establishing CYP8B1 as a key link between BA structure and systemic metabolic signaling phenotypes [306, 307].

Preclinically, CYP8B1 deficiency or knockdown improves metabolic parameters by reducing CA-dependent lipid emulsification, promoting distal fatty acid absorption, and stimulating L-cell GLP-1 secretion, thereby enhancing glucose tolerance and insulin sensitivity [308]. Concurrently, BA composition shifts toward hydrophilicity, increasing fecal lipid and BA excretion, preventing cholesterol-driven NAFLD, and blocking hypercholesterolemia in models of hepatic insulin signaling defects and type 1 diabetes [309]. In humans, CYP8B1 loss-of-function carriers show reduced 12α-hydroxylated BA proportions [310] and require higher glucose infusion rates during hyperinsulinemic-euglycemic clamps, confirming enhanced peripheral insulin sensitivity [307]. Mechanistically, the altered CDCA/CA ratio strengthens insulin signaling and glucose uptake via the CDCA–FOXO1 axis. Given CYP8B1 is non-essential for viability, its inhibition offers a safe therapeutic strategy. Small-molecule inhibitors (e.g., MO-10 analogs) and indirect modulators (e.g., thyroid hormone analogs) are currently under development, representing promising approaches to remodel BA signaling for metabolic disease treatment.

### Hydrophilic and low-toxicity remodeling of the BA signaling environment

This strategy restores physiological BA signaling by replacing hydrophobic, cytotoxic ligands with hydrophilic, low-toxicity species. It simultaneously restores feedback inhibition on upstream synthetic enzymes by supplementing physiological BA end products, thereby reducing toxic intermediate accumulation and limiting pathological receptor or membrane stress signals.

UDCA increases the proportion of hydrophilic BAs, diluting and displacing hydrophobic species, stabilizing cell membranes and mitochondria, and inhibiting BA-induced apoptosis and oxidative stress. It remains first-line therapy for PBC and is used for cholesterol gallstone dissolution and prevention in high-risk populations [311–314]. Its derivative norUDCA, with a shortened C23 side chain, induces bicarbonate-rich hypercholeresis via cholangio-hepatic shunting, exerting anti-cholestatic, anti-inflammatory, and anti-fibrotic effects [315, 316]. NorUDCA significantly improves biochemical markers in PSC but has not yet achieved standard treatment status [317, 318].

Beyond displacement, direct BA supplementation restores feedback inhibition on CYP7A1 and reduces toxic intermediate generation. CA supplementation replenishes deficient primary BAs, improving metabolite profiles, liver function, and fat-soluble vitamin absorption in primary BA synthesis disorders; its formulation CHOLBAM has received FDA approval [319, 320]. In Zellweger spectrum disorders, CA as adjunctive therapy improves hepatic phenotypes and reduces abnormal BA intermediates [321, 322]. For cerebrotendinous xanthomatosis (CTX) caused by CYP27A1 deficiency, CDCA restores physiological feedback inhibition, reducing cholesterol conversion to toxic metabolites (e.g., cholestanol) [323, 324]. Early intervention halts neurological damage progression, and its formulation chenodiol has been approved for adult CTX treatment. However, a limitation of this hydrophilic remodeling strategy lies in its "passive" mode of action—it dilutes toxic species but fails to correct underlying pathological targets (e.g., sustained FXR suppression or aberrant TGR5 activation) driving disease progression. Future therapies must combine hydrophilic BA replacement with targeted receptor modulators to restore BA pool composition and dysregulated signaling networks.

## Conclusions and future directions

BAs are multi-functional signaling molecules with their biological effects depending on the ligand structure, local exposure, tissue receptor expression, microbial modification and receptor selectivity. Through FXR, TGR5, VDR, PXR, CAR, S1PR2, MRGPRX4, and non-receptor mechanisms, BAs regulate metabolism, immunity, epithelial integrity and systemic homeostasis, whereas their dysregulation causes liver and biliary tract, gastrointestinal, metabolic, cardiovascular and malignant diseases. Present therapeutic approaches targeting FXR, TGR5, FGF19, transporters, synthetic enzymes or BA ligand modifications show promise for various indications.

However, several limitations constrain their clinical translation: (1) BA signaling exhibits marked tissue and receptor specificity, leading to off-target effects during systemic regulation (e.g., FXR-mediated LDL elevation and TGR5-mediated gallbladder filling); (2) individual variability in gut microbiota-dependent BA transformation makes it difficult to predict changes in therapeutic BA ligands; thus, metagenomics-guided personalized BA interventions, in which clinicians stratify patients and tailor strategies based on the individual microbial BA-transforming capacity (e.g., 7α-dehydroxylation versus deconjugation activities), such as selective bile salt hydrolase inhibitors or probiotic supplements, to restore each patient's BA signaling environment, should be considered; (3) there are insufficient long-term safety data, especially concerning chronic BA redistribution, cardiovascular risks, and neuroendocrine effects; and (4) the multifaceted nature of MASH/NASH, PSC and cholestatic diseases is beyond the scope of single-target BA-modulating agents.

Previous clinical trial designs in the BA field have generally assumed a homogeneous BA pool across all enrolled patients. The stratified regulatory framework presented in this paper represents the first systematic effort to fundamentally challenge this long-held incorrect assumption. Future research should therefore focus on precise BA profiling to distinguish different patient groups, microbiota-based concurrent interventions to improve ligand control, tissue-specific receptor modifiers to reduce systemic side effects, and reasonable combination regimens (e.g., FXR agonist plus ASBT inhibitor) and appropriate disease models which mimic the complex BA signaling network. Overcoming these issues will be essential for advancing the next generation of targeted and personalized BA-based therapies.

## Data Availability

Not applicable.

## Abbreviations

ACC: Acetyl-CoA carboxylase
AE2: Anion exchanger 2
ALD: Alcohol-associated liver disease
ALP: Alkaline phosphatase
APC: Antigen-presenting cell
AS: Atherosclerosis
ASBT: Apical sodium-dependent bile acid transporter
BA: Bile acid
BAAT: Bile acid-CoA: amino acid N-acyltransferase
BACS: Bile acyl-CoA synthetase
BAD: Bile acid diarrhea
BDL: Bile duct ligation
BSEP: Bile salt export pump
BSH: Bile salt hydrolase
CA: Cholic acid
CAR: Constitutive androstane receptor
CCA: Cholangiocarcinoma
CD: Crohn's disease
CDCA: Chenodeoxycholic acid
CFTR: Cystic fibrosis transmembrane conductance regulator
CGRP: Calcitonin gene-related peptide
COX-2: Cyclooxygenase-2
CRC: Colorectal cancer
CTX: Cerebrotendinous xanthomatosis
CYP7A1: Cholesterol 7α-hydroxylase
CYP8B1: Sterol 12α-hydroxylase
CYP27A1: Sterol 27-hydroxylase
DCA: Deoxycholic acid
DCs: Dendritic cells
DIO2: Type 2 deiodinase
EGFR: Epidermal growth factor receptor
EMT: Epithelial–mesenchymal transition
EPAC: Exchange protein directly activated by cAMP
ERS: Endoplasmic reticulum stress
FGF15/19: Fibroblast growth factor 15/19
FGFR4: Fibroblast growth factor receptor 4
FXR: Farnesoid X receptor
GLP-1: Glucagon-like peptide-1
GPCR: G protein-coupled receptor
HCC: Hepatocellular carcinoma
HDAC1/2: Histone deacetylase 1/2
HNF4α: Hepatocyte nuclear factor 4α
HOMA-IR: Homeostatic model assessment of insulin resistance
HSDHs: Hydroxysteroid dehydrogenases
IBABP: Ileal bile acid-binding protein
IBD: Inflammatory bowel disease
IBS-D: Diarrhea-predominant irritable bowel syndrome
ISC: Intestinal stem cell
LCA: Lithocholic acid
LDL: Low-density lipoprotein
LRH-1: Liver receptor homolog-1
LXRα: Liver X receptor α
MASLD: Metabolic dysfunction-associated steatotic liver disease
MASH: Metabolic dysfunction-associated steatohepatitis
MCAs: Muricholic acids
MMP-9: Matrix metalloproteinase-9
MRGPRX4: MAS-related G protein-coupled receptor X4
MRP2/3/4: Multidrug resistance-associated protein 2/3/4
NAFLD: Nonalcoholic fatty liver disease
NASH: Nonalcoholic steatohepatitis
Nrf2: Nuclear factor erythroid 2-related factor 2
NTCP: Sodium-dependent taurocholate cotransporting polypeptide
OATPs: Organic anion transporting polypeptides
OCA: Obeticholic acid
OSTα/β: Organic solute transporter α/β
PBC: Primary biliary cholangitis
PFIC5: Progressive familial intrahepatic cholestasis type 5
PSC: Primary sclerosing cholangitis
PXR: Pregnane X receptor
PYY: Peptide YY
ROS: Reactive oxygen species
RXR: Retinoid X receptor
S1P: Sphingosine-1-phosphate
S1PR2: Sphingosine-1-phosphate receptor 2
SeHCAT: Selenium homocholic acid taurine
SHP: Small heterodimer partner
SphK2: Sphingosine kinase 2
SULT2A1: Sulfotransferase family 2A member 1
T2DM: Type 2 diabetes mellitus
TCA: Taurocholic acid
TFEB: Transcription factor EB
TGR5: Takeda G protein-coupled receptor 5
Treg: Regulatory T cell
TUDCA: Tauroursodeoxycholic acid
UC: Ulcerative colitis
UDCA: Ursodeoxycholic acid
UGT: UDP-glucuronosyltransferase
VCAM-1: Vascular cell adhesion molecule-1
VDR: Vitamin D receptor
